# Supplementary material for: A net-zero emissions strategy for China’s power sector using carbon-capture utilization and storage
Source: Nat Commun. 2023 Sep 25;14:5972. doi: 10.1038/s41467-023-41548-4 (PMC10520018; doi:10.1038/s41467-023-41548-4)
Supplement: Supplementary file 1 — Supplementary Information [file 41467_2023_41548_MOESM1_ESM.pdf]

*Supplementary Information for*

# A net-zero emissions strategy for China's power sector using carbon capture utilization and storage

Jing-Li Fan, Zezheng Li, Xi Huang, Kai Li, Xian Zhang, Xi Lu, Jian Zhong Wu, Klaus Hubacek, Bo Shen

## Supplementary Figures

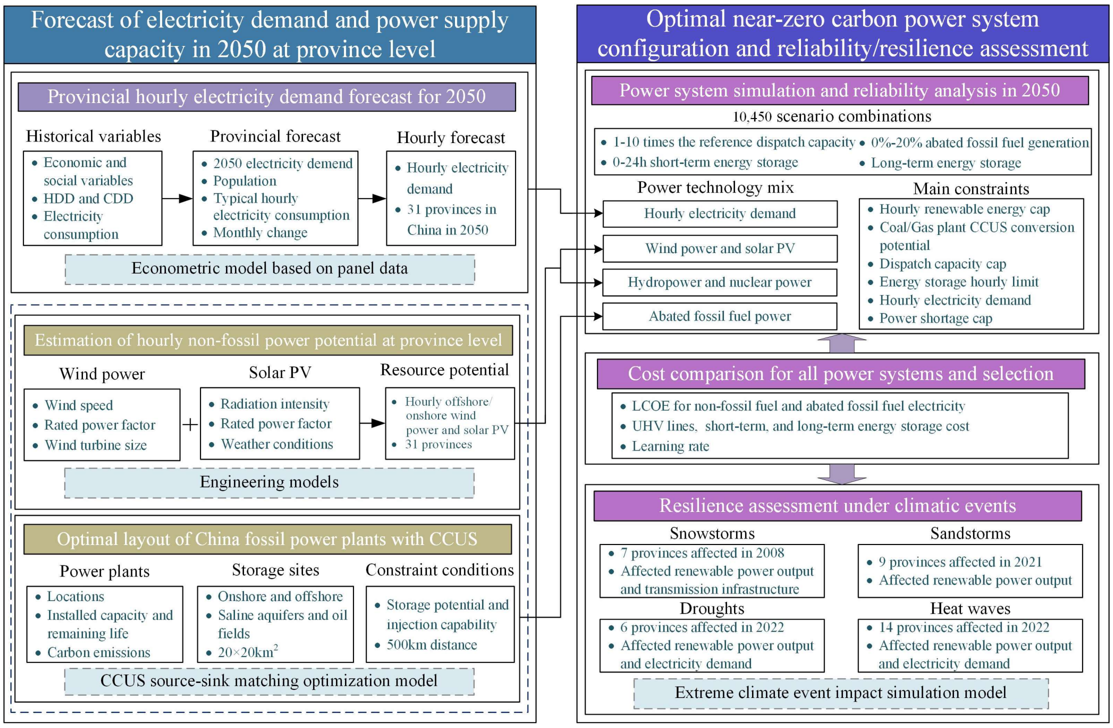


**Supplementary Figure 1. Research framework.** The left large box represents the forecasting of electricity demand and supply potential and the right one represents the near-zero carbon power system models and simulations., Specifically, the left side of the figure includes an hour-by-hour prediction model for electricity demand for 31 Chinese provinces in 2050, an hour-by-hour estimation model for solar PV and wind power generation potential for 31 Chinese provinces, and a CCUS source-sink optimal matching model for retrofitting China's existing fossil fuel power plants. The right side of the figure includes an integrated simulation model configured with hourly power system supply-demand balance in 31 provinces in 2050, a cost-competitive analysis of the decarbonized power system in 2050, which is used to identify the lowest-cost power mix, and the simulation model of the impacts of representative weather extremes (snowstorms, sandstorms, droughts and heat waves) on power generation and corresponding supply shortage, which is used to assess the resilience of future power system.

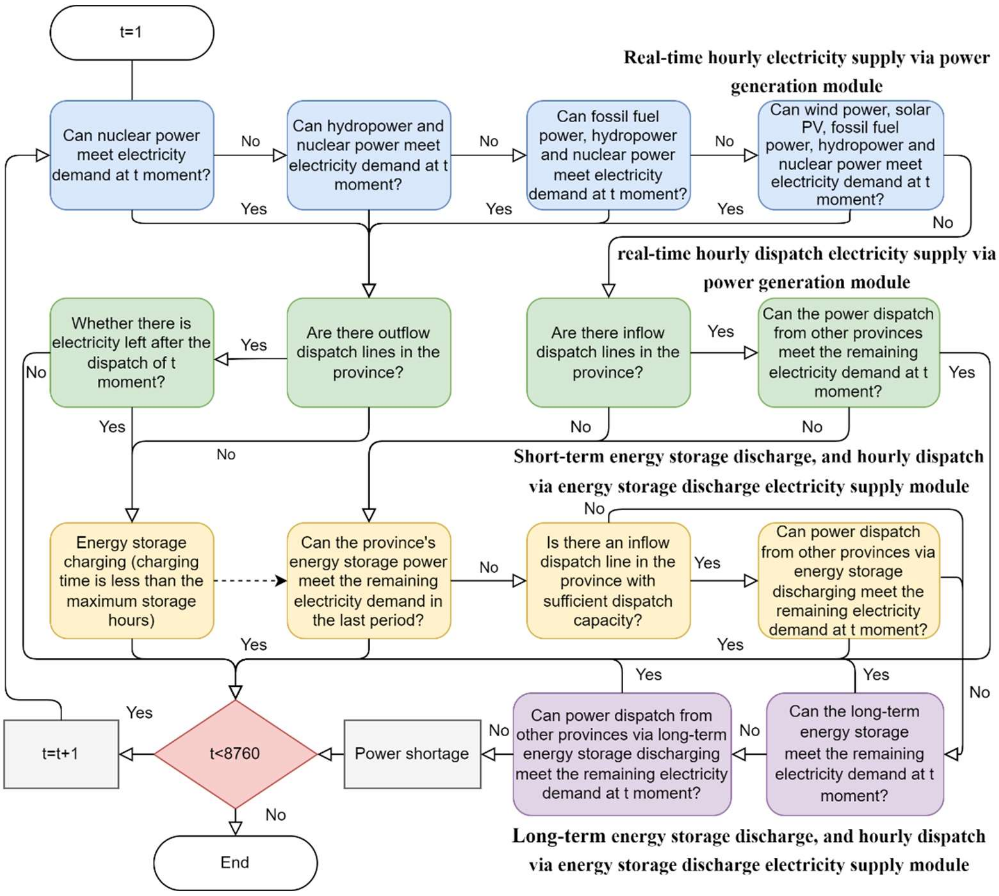


**Supplementary Figure 2. Optimization flowchart for China's near-zero power system in 2050.** The blue areas represent local real-time hourly electricity supply via power generation module, the green areas represent real-time hourly dispatch electricity supply via power generation module, the yellow areas represent short-term energy storage discharge and hourly dispatch via energy storage discharge electricity supply module, the purple areas represent long-term energy storage discharge and hourly dispatch via energy storage discharge electricity supply module, the diamond box represents the loop condition, the solid lines represent the real-time hourly situation at the current moment, and the dashed line represents the local energy storage at the previous moment for discharge at moment *t*.

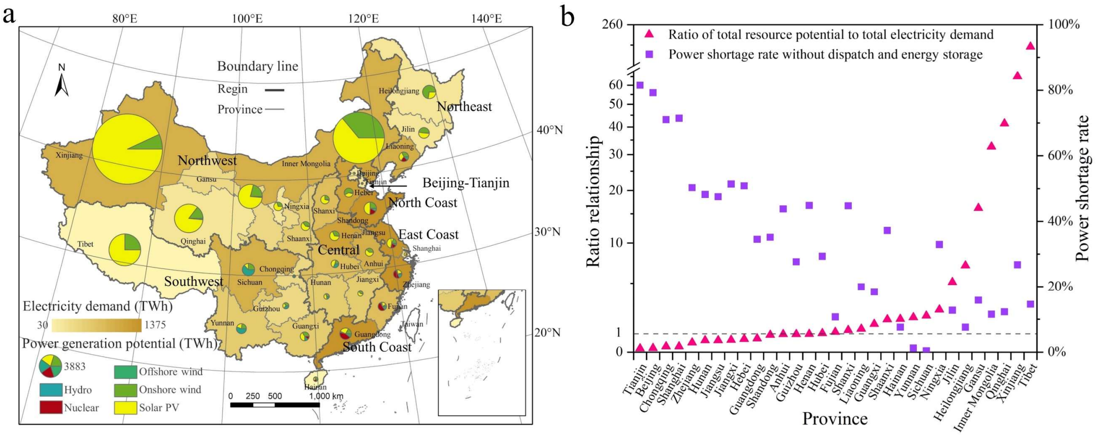


**Supplementary Figure 3. Comparison between non-fossil fuel power generation potential and electricity demand by province in 2050. a** Distribution of non-fossil fuel power generation potential and electricity consumption by province; wind power and solar PV generation potential is determined by summing up the real-time hourly power output. **b** Values of two gap indicators between non-fossil power generation potential and electricity demand; red triangles represent the ratio of total non-fossil power generation potential to total electricity consumption in each province (all hours cumulative); purple squares represent the provincial total power shortage rate when electricity supply relying solely on non-fossil fuel without transmission and energy storage. Data Credits: All the provincial boundaries are from Ministry of Civil Affairs of the People's Republic of China (http://xzqh.mca.gov.cn/map, Map Content Approval Number: GS (2022)1873).

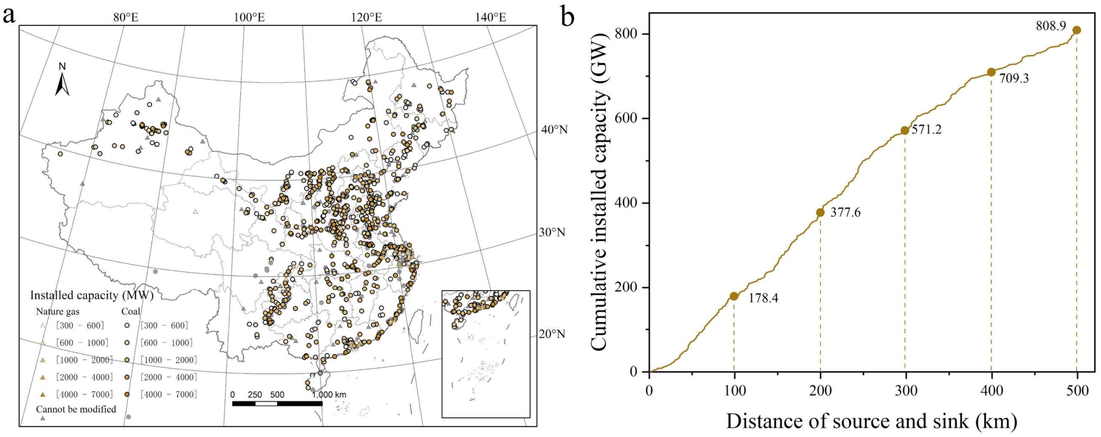


**Supplementary Figure 4. Distribution of fossil fuel power plants and CCUS supply curve in China. a** Distribution of coal-fired and gas-fired power plants in China, where triangles represent gas-fired power plants, circles represent coal-fired power plants, color shades represent installed capacity, and gray ones represent power plants that do not meet CCUS retrofit standards. **b** The relationship between source-sink distance and cumulative installed capacity of CCUS retrofit after source-sink matching. Data Credits: All the provincial boundaries are from Ministry of Civil Affairs of the People's Republic of China (http://xzqh.mca.gov.cn/map, Map Content Approval Number: GS (2022)1873).

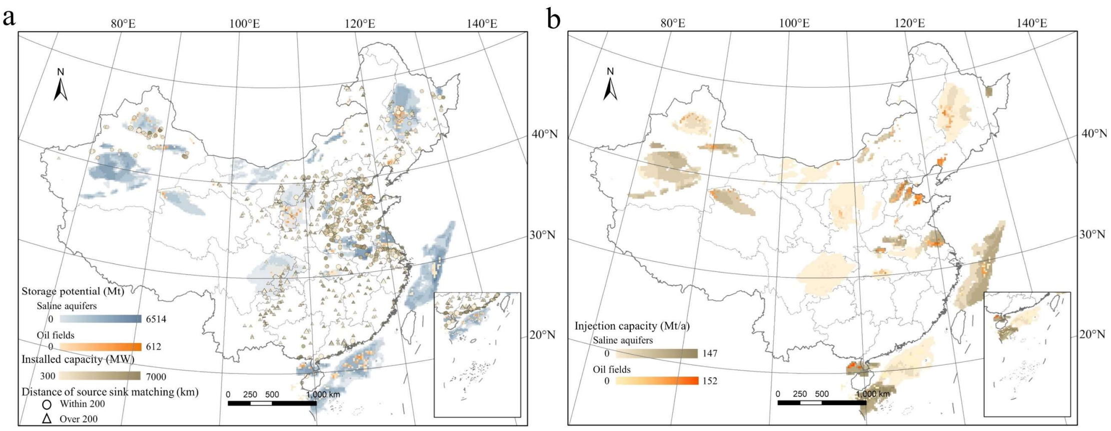


**Supplementary Figure 5. Spatial distribution of CCUS source-sink matching results associated with existing power plants, storage sites and matching distances** [1, 2]**. a** Distribution of CCUS retrofitted plants, saline aquifers, and oil fields, as well as their grid-scale storage capacity. The circles/triangles identify fossil fuel power plants that match storage sites within/without 200 km with color shades representing installed capacity. **b** Distribution pattern of injection capacity at storage sites. Data Credits: All the provincial boundaries are from Ministry of Civil Affairs of the People's Republic of China (http://xzqh.mca.gov.cn/map, Map Content Approval Number: GS (2022)1873).

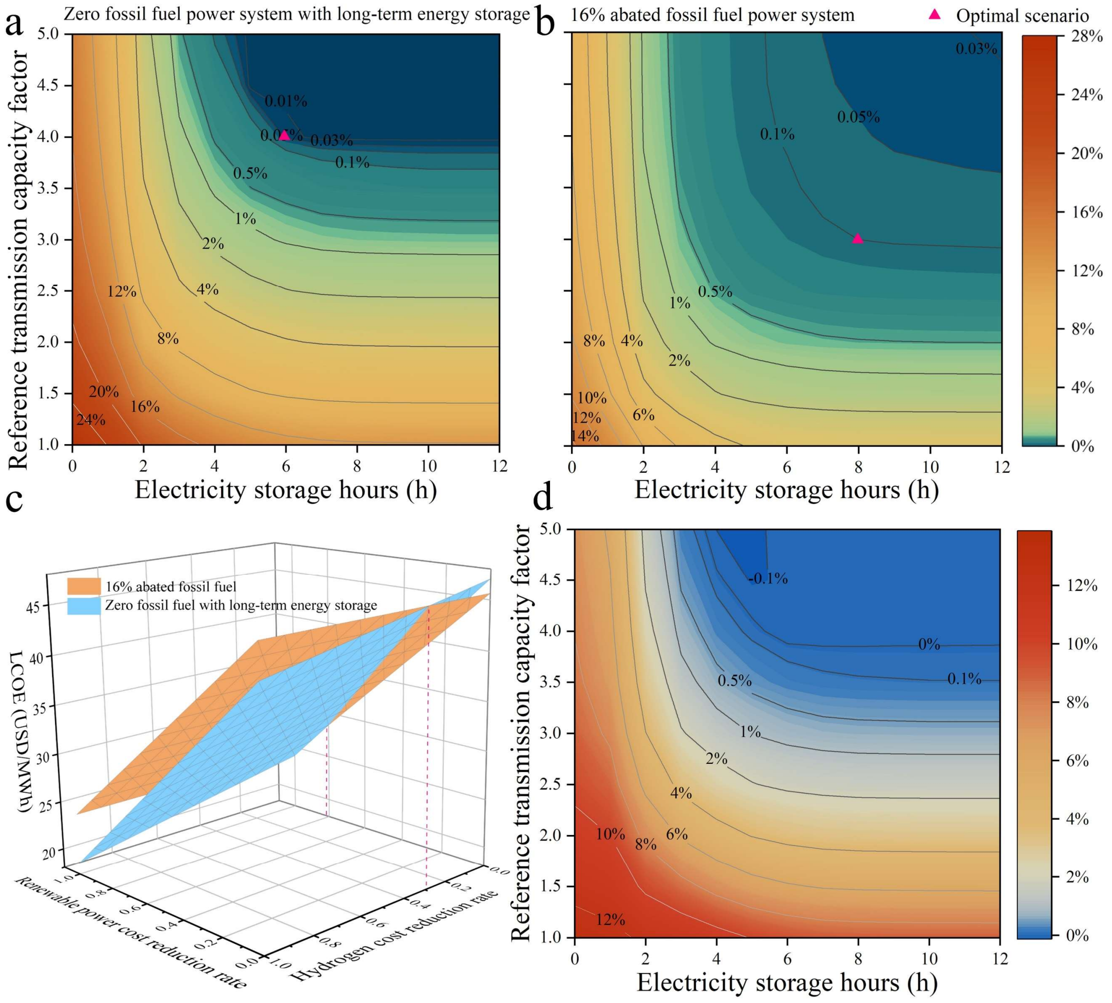


**Supplementary Figure 6. Power shortage and cost comparison under 16% abated fossil fuel and zero fossil fuel with long-term energy storage scenarios.** Power shortage rates for various combination scenarios of transmission capacity and energy storage hours: **a** zero fossil fuel with long-term energy storage; **b** 16% abated fossil fuel. A warmer color indicates a more severe power shortage, while a cooler color indicates a less severe shortage. The lines represent the combination of transmission capacity and energy storage hours for a specific level of power shortage. The red triangle represents the minimum cost required to meet the supply reliability criteria for the given set of scenarios. **c** Cost comparison under different scenarios in which the cost of variable renewable energy (wind power and solar PV) and hydrogen (hydrogen production, storage, and power generation) decreases. **d** Power shortage rates gap between the zero fossil fuel with long-term energy storage scenario and the 16% abated fossil fuel scenario at various combinations of transmission capacity and energy storage hours.

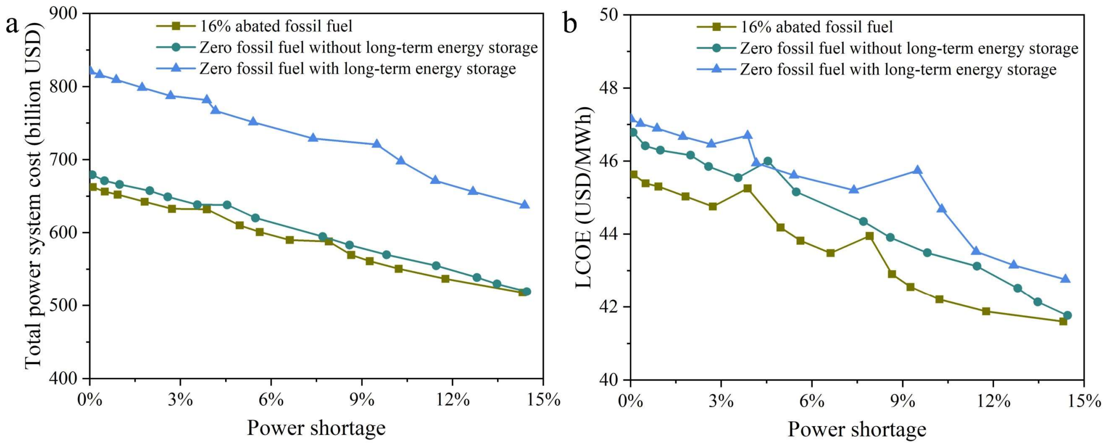


**Supplementary Figure 7. Comparison of the total cost and the LCOE of power system under 16% abated fossil fuel and zero fossil fuel with or without long-term energy storage scenarios. a** Total cost of power system; **b** LCOE of power system.

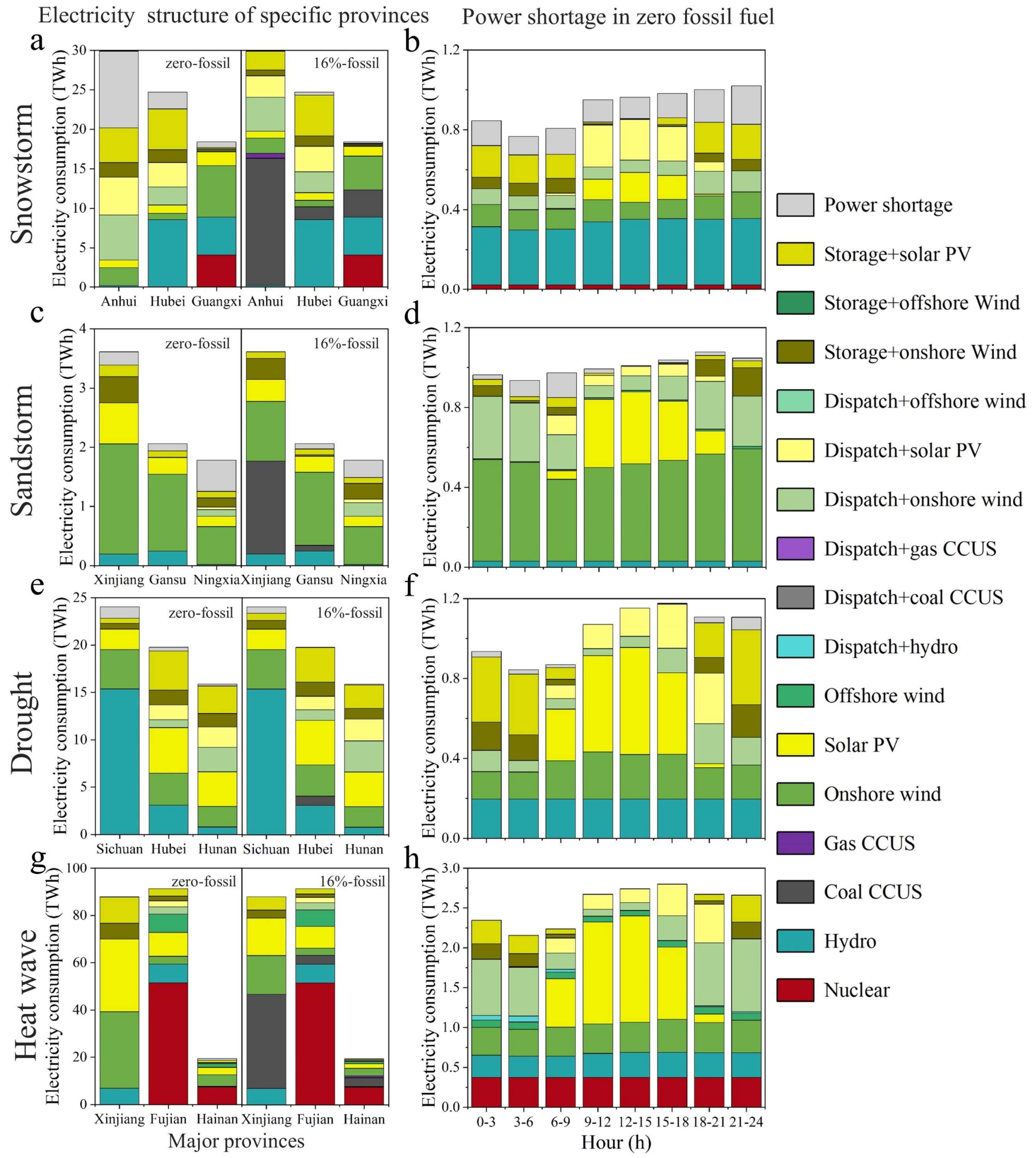


**Supplementary Figure 8. Electricity structure and the impact of disasters on the power shortage in major affected provinces under two scenarios.** The left column represents the electricity consumption structure of specific provinces during snowstorm events (**a**), sandstorm events (**c**), drought events (**e**), and heat wave events (**g**), respectively; the right column represents the power shortage and electricity consumption structure at aggregated 3-hour intervals under zero fossil fuel scenario during snowstorm events (**b**), sandstorm events (**d**), drought events (**f**), and heat wave events (**h**), respectively.

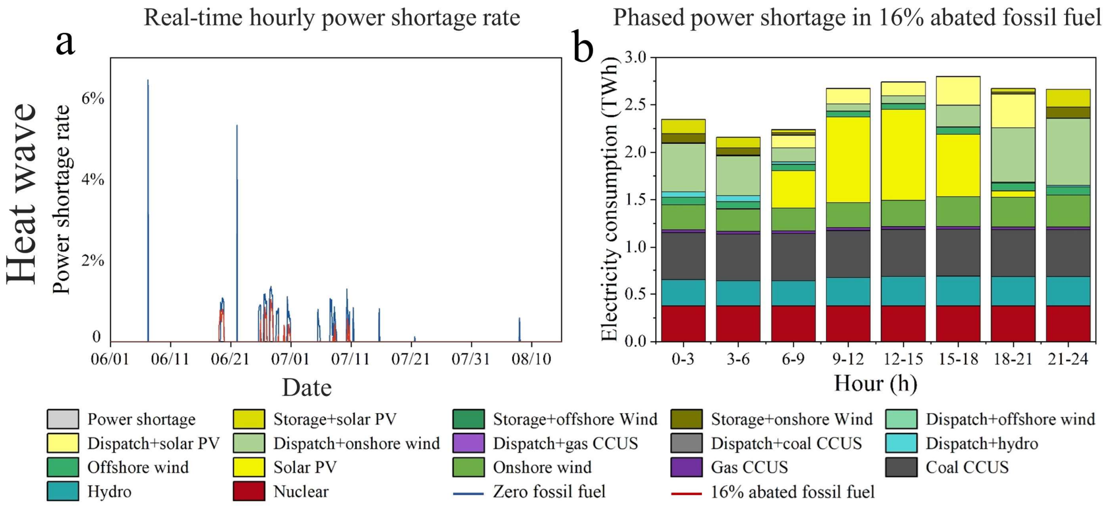


**Supplementary Figure 9. Comparison of power shortage in affected provinces during heat wave events. a** The real-time power shortage rates. **b** The composition of electricity consumption in a 3-hour cycle. Note that the event occurrence time, duration, intensity, and affected provinces are sourced from actual heat wave disasters in China from June 1 to August 31, 2022.

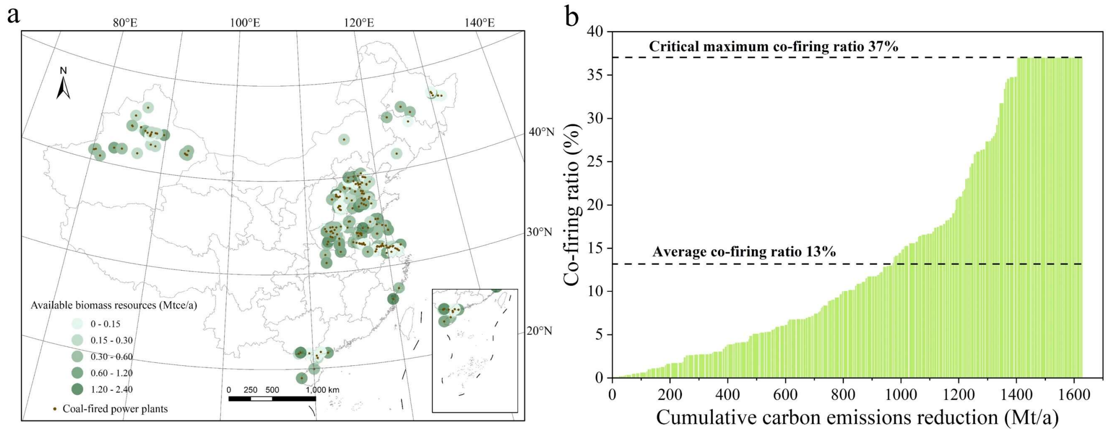


**Supplementary Figure 10. Biomass feedstocks surrounding CCUS-qualified fossil fuel power plants and corresponding actual co-firing ratios. a** The spatial distribution of available biomass feedstocks delivered to related power plants within 50 km. **b** The relationship between the cumulative carbon emissions reduction of various power plants and their actual co-firing ratios. The overall fossil fuel power plants can achieve net-zero emissions when the maximum co-firing ratio hits 37% and the national average co-firing ratio reaches 13%. Data Credits: All the provincial boundaries are from Ministry of Civil Affairs of the People's Republic of China (http://xzqh.mca.gov.cn/map, Map Content Approval Number: GS (2022)1873).

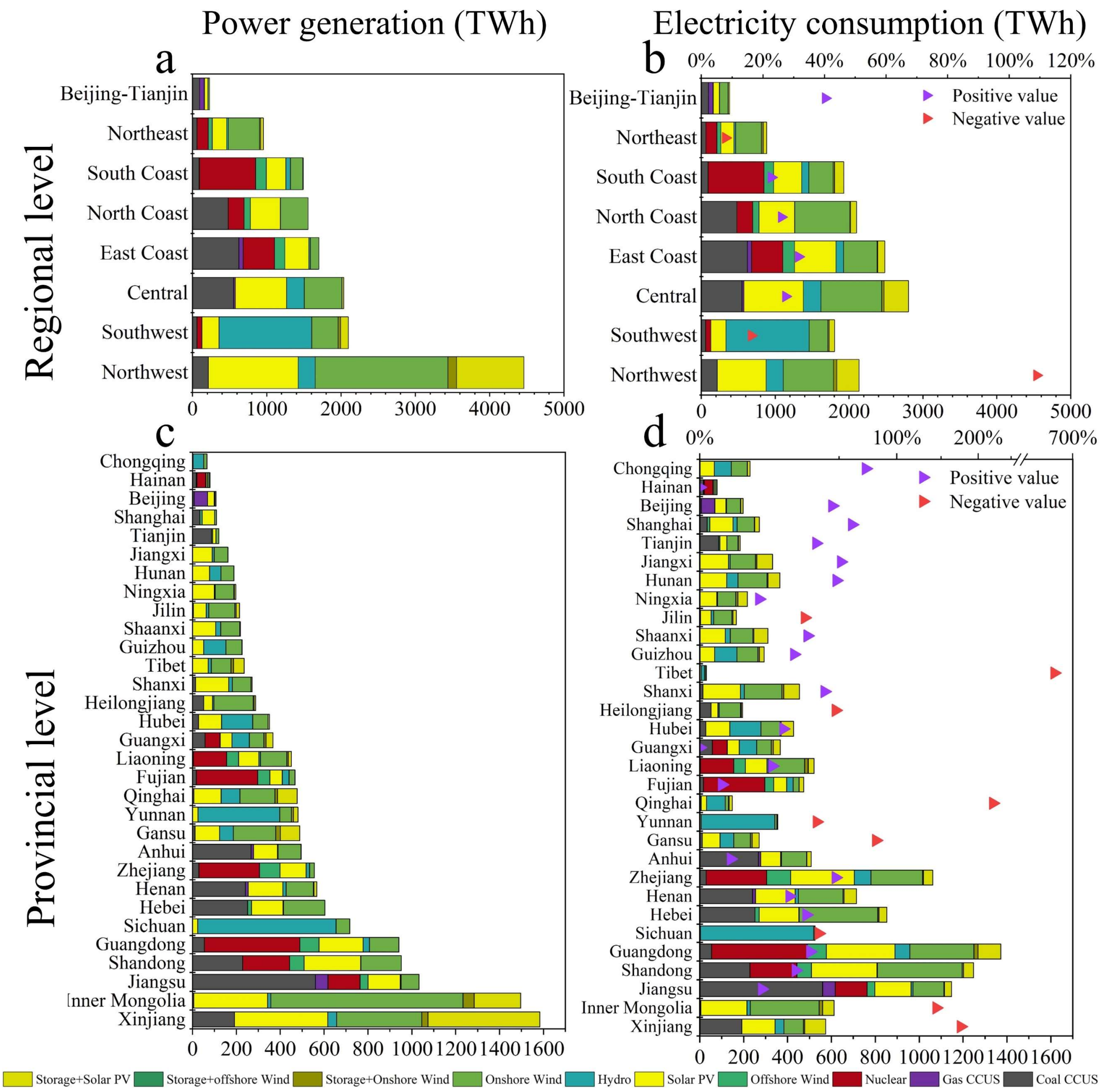


**Supplementary Figure 11. Comparison of power generation and electricity consumption at the regional and provincial levels. a** Regional power generation. **b** Regional electricity consumption. **c** Provincial power generation. **d** Provincial electricity consumption. The purple and red triangles indicate positive and negative external dependencies for each region and province, respectively.

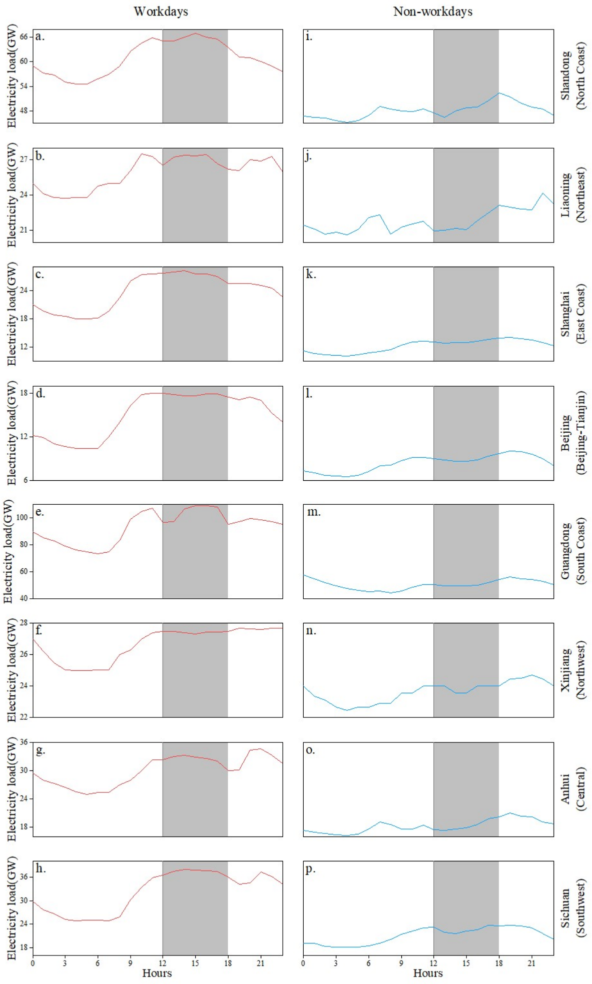


**Supplementary Figure 12. Hourly electricity load curves for typical workdays and non-workdays in representative provinces of eight regions [3].**

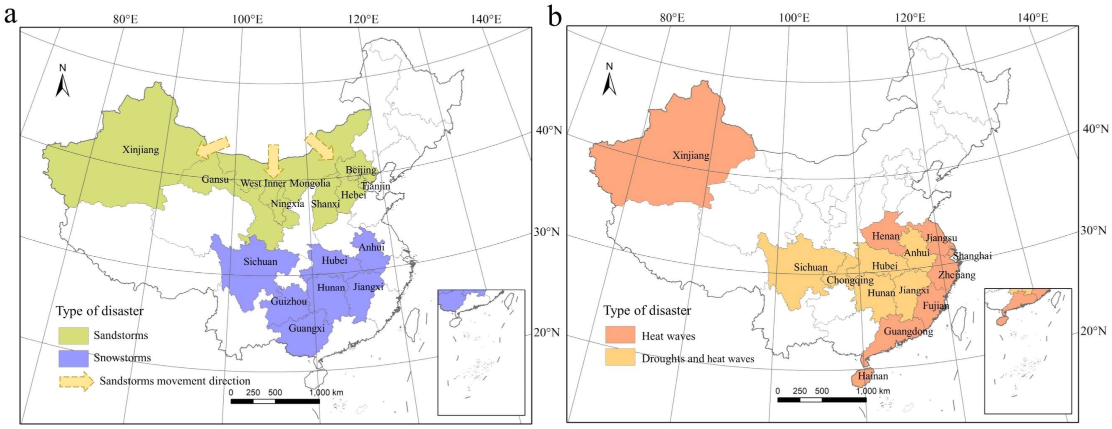


**Supplementary Figure 13. China's major affected provinces during various extreme climatic events. a** The southern snowstorm in 2008 and the north sandstorm in 2021. **b** The drought in 2022 and the heat wave in 2022. Data Credits: All the provincial boundaries are from Ministry of Civil Affairs of the People's Republic of China (http://xzqh.mca.gov.cn/map, Map Content Approval Number: GS (2022)1873).

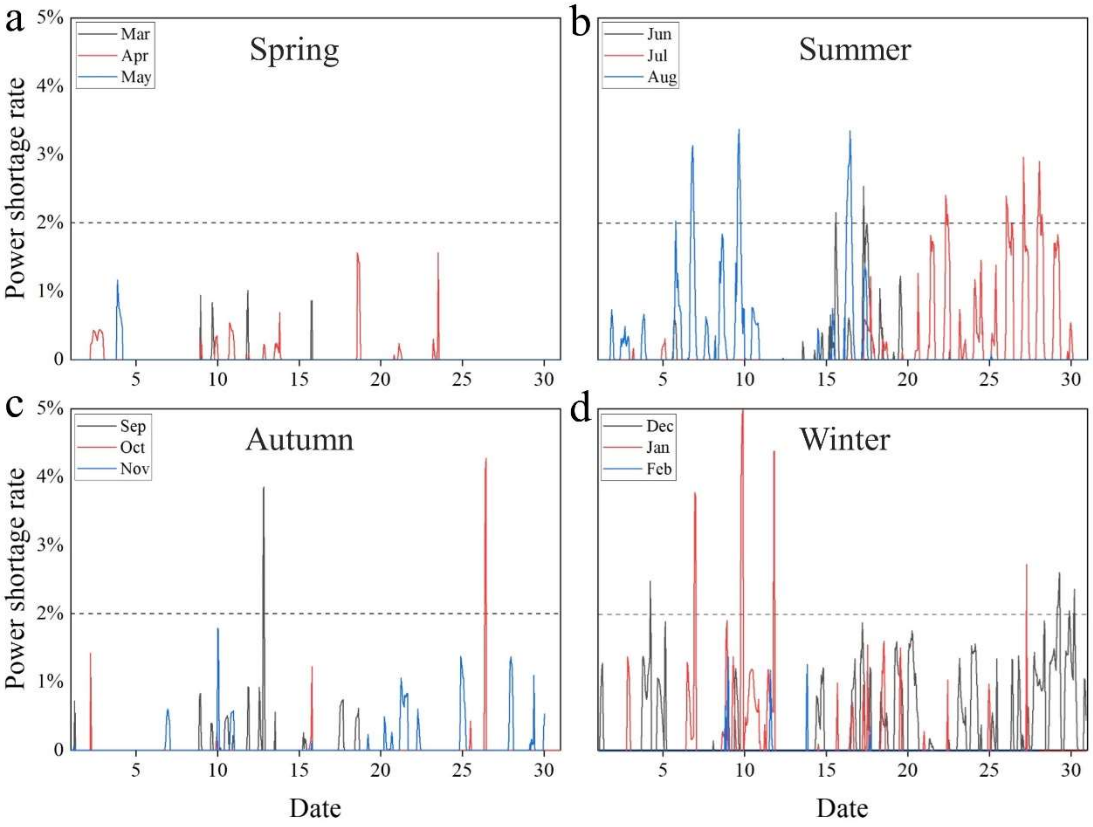


**Supplementary Figure 14. Hourly variation in the national four-season power shortage rate under the 16% abated fossil fuel scenario.** Each panel represents a different season: **a** spring (March, April, May); **b** summer (June, July, August); **c** autumn (September, October, November); **d** winter (December, January, February). The dotted line represents those hours when the hourly power shortage rate exceeds 2%.

# Supplementary Tables

**Supplementary Table 1. Existing and supposed transmission routes in the reference case**

| Existing transmission routes | | | New transmission routes | | |
|---|---|---|---|---|---|
| Inflow | Outflow | Capacity (GW) | Inflow | Outflow | Capacity (GW) |
| Beijing | Hebei | 6 | Guangdong | Tibet | 8 |
| Tianjin | Western Inner Mongolia | 6 | Guangdong | Qinghai | 8 |
| Hebei | Shanxi | 7 | Guangdong | Xinjiang | 8 |
| Hebei | Eastern Inner Mongolia | 4.8 | Zhejiang | Western Inner Mongolia | 8 |
| Hebei | Western Inner Mongolia | 20.92 | Zhejiang | Xinjiang | 8 |
| Hebei | Liaoning | 3 | Zhejiang | Qinghai | 8 |
| Hebei | Shandong | 4 | Jiangsu | Qinghai | 8 |
| Hebei | Shaanxi | 6.24 | Jiangsu | Xinjiang | 8 |
| Shanghai | Jiangsu | 10 | Jiangsu | Gansu | 8 |
| Shanghai | Zhejiang | 5 | Beijing | Western Inner Mongolia | 8 |
| Shanghai | Anhui | 20 | Beijing | Heilongjiang | 8 |
| Shanghai | Hubei | 10.164 | Jiangxi | Xinjiang | 8 |
| Shanghai | Sichuan | 6.4 | Jiangxi | Qinghai | 8 |
| Jiangsu | Shanxi | 8 | Shanxi | Xinjiang | 8 |
| Jiangsu | Western Inner Mongolia | 10 | Shanxi | Western Inner Mongolia | 8 |
| Jiangsu | Hubei | 3 | Anhui | Western Inner Mongolia | 8 |
| Jiangsu | Sichuan | 7.2 | Anhui | Gansu | 8 |
| Jiangsu | Shaanxi | 8 | Shaanxi | Xinjiang | 8 |
| Zhejiang | Anhui | 10 | Shaanxi | Qinghai | 8 |
| Zhejiang | Fujian | 16.8 | Guizhou | Tibet | 8 |
| Zhejiang | Sichuan | 8 | Guizhou | Qinghai | 8 |
| Zhejiang | Ningxia | 8 | Hebei | Heilongjiang | 8 |
| Anhui | Xinjiang | 12 | Hebei | Jilin | 8 |
| Fujian | Zhejiang | 6.8 | Tianjin | Eastern Inner Mongolia | 8 |
| Jiangxi | Hubei | 10 | Tianjin | Jilin | 8 |
| Shandong | Hebei | 8 | Shanghai | Xinjiang | 8 |
| Shandong | Eastern Inner Mongolia | 10 | Shanghai | Western Inner Mongolia | 8 |
| Shandong | Western Inner Mongolia | 16 | Henan | Western Inner Mongolia | 8 |
| Shandong | Shaanxi | 6 | Hunan | Xinjiang | 8 |
| Shandong | Gansu | 8 | Fujian | Xinjiang | 8 |
| Shandong | Ningxia | 4 | Liaoning | Eastern Inner Mongolia | 8 |
| Henan | Shanxi | 10 | Hubei | Xinjiang | 8 |
| Henan | Hubei | 5 | Shandong | Xinjiang | 8 |

| Existing transmission routes | | | New transmission routes | | |
|---|---|---|---|---|---|
| Inflow | Outflow | Capacity (GW) | Inflow | Outflow | Capacity (GW) |
| Henan | Chongqing | 2.5 | Ningxia | Gansu | 8 |
| Henan | Shaanxi | 1.11 | Chongqing | Tibet | 8 |
| Henan | Qinghai | 8 | | | |
| Henan | Xinjiang | 8 | | | |
| Hubei | Henan | 15.5 | | | |
| Hubei | Chongqing | 2.5 | | | |
| Hubei | Shaanxi | 8 | | | |
| Hunan | Hubei | 10 | | | |
| Hunan | Gansu | 8 | | | |
| Guangdong | Hubei | 5 | | | |
| Guangdong | Guizhou | 5 | | | |
| Guangdong | Yunnan | 15 | | | |
| Chongqing | Sichuan | 5.5 | | | |
| Chongqing | Xinjiang | 8 | | | |
| Sichuan | Shaanxi | 3 | | | |
| Tibet | Sichuan | 3 | | | |
| Tibet | Qinghai | 0.6 | | | |

**Supplementary Table 2. Current maturity and cost of various types of energy storage technologies in China** [4, 5, 6, 7, 8]

| Types of energy storage | Technical classification | Technology maturity | Cost |
|---|---|---|---|
| Thermal energy storage | Cold and energy storage | H | H |
| | Thermal energy storage | H | M |
| Mechanical energy storage | Pumped hydroelectric storage | EH | L |
| | Compressed air energy storage | H | M |
| | Flywheel energy storage | M | H |
| Electromagnetic energy storage | Ultracapacitor energy storage | M | H |
| | Superconductor energy storage | L | EH |
| Electrochemical energy storage | Li-ion battery | H | M |
| | Sodium-ion battery | M | H |
| | Lead-acid battery | H | M |
| | Liquid flow battery | M | H |
| Chemical energy storage | Hydrogen energy storage | M | EH |

Note: The maturity and cost intensity of the technology includes four levels: Low (L), Moderate (M), High (H), and Extremely high (EH).

**Supplementary Table 3. Power system costs under the zero fossil fuel scenario and the 16% abated fossil fuel scenario**

| Total cost (billion USD) | | Transmission capacity | | | | | | | | | |
|---|---|---|---|---|---|---|---|---|---|---|---|
| Energy storage hours | | 3 | | 3.5 | | 4 | | 4.5 | | 5 | |
| | | Zero fossil fuel | 16% abated fossil fuel | Zero fossil fuel | 16% abated fossil fuel | Zero fossil fuel | 16% abated fossil fuel | Zero fossil fuel | 16% abated fossil fuel | Zero fossil fuel | 16% abated fossil fuel |
| 5 | TT | -- | -- | -- | -- | -- | -- | -- | -- | -- | -- |
| | DS&ES | -- -- | -- -- | -- -- | -- -- | -- -- | -- -- | -- -- | -- -- | -- -- | -- -- |
| 6 | TT | -- | -- | -- | -- | -- | 669.1 | -- | 673.1 | -- | 676.9 |
| | DS&ES | -- -- | -- -- | -- -- | -- -- | -- -- | 44.2 47.7 | -- -- | 49.2 46.6 | -- -- | 53.7 45.8 |
| 7 | TT | -- | -- | -- | 665.7 | -- | 669.4 | 682.5 | 673.3 | 686.0 | 677.1 |
| | DS&ES | -- -- | -- -- | -- -- | 39.0 49.5 | -- -- | 44.2 47.9 | 49.9 72.3 | 49.1 46.8 | 54.7 70.9 | 53.7 45.9 |
| 8 | TT | -- | 662.4 | -- | 665.7 | 679.2 | 669.4 | 682.5 | 673.3 | 686.0 | 677.2 |
| | DS&ES | -- -- | 33.7 51.9 | -- -- | 39.0 49.6 | 44.8 74.3 | 44.2 48.0 | 49.9 72.3 | 49.1 46.8 | 54.7 70.9 | 53.7 46.0 |
| 9 | TT | -- | 662.5 | -- | 665.8 | 679.2 | 669.4 | 682.5 | 673.3 | 686.0 | 677.2 |
| | DS&ES | -- -- | 33.7 51.9 | -- -- | 39.0 49.7 | 44.8 74.3 | 44.2 48.0 | 49.9 72.4 | 49.1 46.9 | 54.7 70.9 | 53.7 46.0 |
| 10 | TT | -- | 662.5 | -- | 665.8 | 679.2 | 669.4 | 682.5 | 673.4 | 686.0 | 677.2 |
| | DS&ES | -- -- | 33.7 51.9 | -- -- | 39.0 49.7 | 44.8 74.3 | 44.2 48.0 | 49.9 72.4 | 49.1 46.9 | 54.7 70.9 | 53.7 46.1 |
| 11 | TT | -- | 662.5 | -- | 665.8 | 679.2 | 669.5 | 682.5 | 673.4 | 686.0 | 677.2 |
| | DS&ES | -- -- | 33.7 52.0 | -- -- | 39.0 49.7 | 44.8 74.3 | 44.2 48.1 | 49.9 72.4 | 49.1 46.9 | 54.7 71.0 | 53.7 46.1 |
| 12 | TT | -- | 662.5 | -- | 665.8 | 679.2 | 669.4 | 682.5 | 673.4 | 686.0 | 677.2 |
| | DS&ES | -- -- | 33.7 52.0 | -- -- | 39.0 49.7 | 44.8 74.3 | 44.1 48.1 | 49.9 72.4 | 49.1 46.9 | 54.7 71.0 | 53.7 46.1 |

Note: TT represents the total cost in near-zero power system, DS&ES represents the cost of transmission capacity and energy storage in near-zero power system, and ”--” represents the power shortage of the power system in this scenario exceeds 0.1%.

# Supplementary Notes

## Supplementary Note 1 Assessment of non-fossil fuel power potential

The approach for assessing the generation potential of five non-fossil power sources, including onshore wind power, offshore wind power, solar PV, hydropower, and nuclear power, is introduced in this note. It should be noted that Figure 1 shows the calculated daily generation potential of wind power and solar PV over the sample years (1980-2019 for wind power and 2010-2019 for solar PV), with an emphasis on the average generation of these years. In order to better reflect the actual intermittency and volatility of wind and solar PV, we selected 2016 as the reference year for wind and PV potential estimation in the power system model because it has the smallest difference to the average value of above generation potential.

### **Supplementary Note 1.1** Wind power potential assessment

Given that earlier studies investigated the wind power potential employing onshore and offshore turbines with rated capacities ranging from 1.5-3.4 and 5-8 MW [9, 10, 11], as well as the newly-added single-unit installed capacity, we have set the rated capacity of onshore wind as 2 MW turbines at 90m hub height, and the rated capacity of offshore wind as 6.45 MW turbines at 108m hub height to represent the average level of wind power in China. Based on the real-time hourly wind speeds in the capital cities of each province in China from 1980 to 2019, the real-time hourly power output of each standard wind turbine onshore or offshore is calculated using the power curve, as expressed in Equation (1).

$$SWP_{n,t} = \begin{cases} 0, & V_{n,t} < V^{ci} \ or \ V_{n,t} > V^{co} \\ \frac{1}{2} C^P \cdot \pi r^2 \cdot \rho \cdot V_{n,t}^3, & V^{ci} \le V_{n,t} \le V^r \\ SWP^R, & V^r < V_{n,t} \le V^{co} \end{cases} \tag{1}$$

where $SWP_{n,t}$ denotes the real-time hourly power output of a single wind turbine at hour $t$ in province $n$ (MW), $V_{n,t}$ denotes the average real-time hourly wind speed (m/s), $V^{ci}$ denotes the cut-in wind speed (m/s), $V^{co}$ denotes the cut-out wind speed (m/s), $C^P$ denotes the power coefficient of the wind turbine, $r$ denotes the impeller radius of the wind turbine (m), $\rho$ denotes the air density (kg/m$^3$), $V^r$ denotes the rated wind speed (m/s), and $SWP^R$ denotes the rated wind turbine power (MW).

The total annual generation potential of wind power in each province from the Renewable Energy Data Book 2019 [12] is then divided by the annual generation potential of a single standard wind turbine to obtain the number of turbines per province. The real-time hourly potential of power output in each province $n$ is then calculated by Equation (2).

$$WP_{n,t} = N_n^W \cdot SWP_{n,t} \tag{2}$$

where $WP_{n,t}$ denotes the real-time hourly wind power output at hour *t* in province *n* (MW), $N_n^W$ denotes the number of wind turbines in province *n*.

The wind power installed capacity potential in each province is determined by the maximum power output of a year, and the wind power generation potential in each province is calculated by the total cumulative power output of each hour in the year, as shown in Equations (3)-(4).

$$WP_n^{IC} = \max WP_{n,t} \tag{3}$$

$$G_n^W = \sum_t WP_{n,t} \tag{4}$$

where $WP_n^{IC}$ denotes the potential of wind power installed capacity of province *n* (MW), $G_n^W$ denotes the annual potential of wind power generation of province *n* (MWh). The potential of installed capacity and annual potential of generation of onshore and offshore wind power in each province are shown in Supplementary Table 4.

## **Supplementary Note 1.2** Solar PV potential assessment

Two methods are used to assess the real-time hourly solar PV output potential, and the aggregated results are compared with the provincial generation potential reported by the National Meteorological Administration [11]. The result with lower deviation is selected as the real-time hourly solar PV output for each province. Here we chose photovoltaic panels with a single fixed-tilt system rather than the more advanced bi-facial or single-axis tracking systems. Although bi-facial systems and single-axis tracking systems theoretically can track the sun completely for higher solar energy output [13], single fixed-tilt systems are more widely used, simpler, cheaper, and have lower maintenance requirements [14]. In this system, adopting an optimal tilt and azimuth angle can help the photovoltaic array receive more solar radiation and increase the overall power generation of the solar PV system [15].

The first method is based on the Hybrid Optimization of Multiple Energy Resources (HOMER) model [16, 17, 18]. By this method, the power output of solar PV panels by comparing standard test conditions with real-time hourly radiation intensity and temperature conditions is calculated by Equation (5).

$$SPV_{n,t}^{V1} = SPV^R \cdot \left( \frac{R_{n,t}}{R^{STC}} \right) \cdot \left[ 1 + \alpha \left( T_{n,t} - T^{STC} \right) \right] \cdot (1 - \gamma) \tag{5}$$

where $SPV_{n,t}^{V1}$ denotes the real-time hourly power output of a single solar PV module at hour *t* in province *n* (W), $SPV^R$ is the rated power of the solar PV module (W), $R_{n,t}$ is the actual solar radiation intensity at hour *t* in province *n* (W/m$^2$), $R^{STC}$ is the radiation intensity at standard test conditions (W/m$^2$), $\alpha$ is the temperature coefficient of the solar PV module (%/℃), $T_{n,t}$ is the actual temperature of the solar PV module at hour *t* in province *n* (℃), $T^{STC}$ is the temperature at standard test conditions (℃), and $\gamma$ is the shading factor of the solar PV array.

The second method is based on different modules and variations in conversion efficiency [19]. This method considers the effects of various solar PV modules and the different performance of conversion efficiency for solar PV systems with varying radiation intensity, as shown in Equation (6).

$$SPV_{i,n,t}^{V2} = \eta_{i,t} \cdot S_i \cdot R_{n,t} \cdot \left[ 1 - 0.005 \cdot \left( T_{n,t} + 25 \right) \right] \tag{6}$$

where $SPV_{i,n,t}^{V2}$ denotes the real-time hourly power output of a single solar PV module $i$ at hour $t$ in province $n$ (W), where $i$=1 is monocrystalline silicon module, $i$=2 is polycrystalline silicon module, and $i$=3 is thin-film solar module; $\eta_{i,t}$ is the conversion efficiency of solar PV module $i$ under different radiation intensity, is calculated by Equation (7) [20], $S_i$ is the area of solar PV module $i$ ($m^2$).

$$\eta_{1,t} = \begin{cases} 3.5781 \cdot \ln R_{n,t} - 4.0401, & 10 \le R_{n,t} \le 200 \\ -0.0006 \cdot R_{n,t} + 14.798, & R_{n,t} > 200 \end{cases}$$
$$\eta_{2,t} = \begin{cases} 3.4155 \cdot \ln R_{n,t} - 5.0545, & 10 \le R_{n,t} \le 200 \\ -0.0012 \cdot R_{n,t} + 12.71, & R_{n,t} > 200 \end{cases} \tag{7}$$
$$\eta_{3,t} = \begin{cases} 1.9076 \cdot \ln R_{n,t} - 3.4561, & 10 \le R_{n,t} \le 200 \\ -0.0003 \cdot R_{n,t} + 6.7909, & R_{n,t} > 200 \end{cases}$$

The total solar PV potential of installed capacity in each province from the National Meteorological Administration [11] is divided by the rated power of a single solar PV module to obtain the number of solar PV modules in each province. Then, the real-time hourly potential of power output in each province $n$ using two methods is calculated by Equations (8)-(9).

$$PV_{n,t}^{V1} = N_n^{V1} \cdot SPV_{n,t}^{V1} \tag{8}$$

$$PV_{n,t}^{V2} = \sum_i N_{i,n}^{V2} \cdot SPV_{i,n,t}^{V2} \tag{9}$$

where $PV_{n,t}^{V1}$ and $PV_{n,t}^{V2}$ denote the real-time hourly power output calculated by the two methods at hour $t$ in province $n$, respectively (W), $N_n^{V1}$ is the number of solar PV panels that can be installed in province $n$, and $N_{i,n}^{V2}$ is the number of solar PV module $i$ that can be installed in province $n$.

Like wind power, the solar PV installed capacity potential in each province is determined by the maximum power output of the year, and the solar PV generation potential in each province is calculated by the total power output of the year, as shown in Equations (10)-(13).

$$PV_n^{IC1} = \max PV_{n,t}^{V1} \tag{10}$$

$$PV_n^{IC2} = \max PV_{n,t}^{V2} \tag{11}$$

$$G_n^{V1} = \sum_t PV_{n,t}^{V1} \tag{12}$$

$$G_n^{V2} = \sum_t PV_{n,t}^{V2} \tag{13}$$

where $PV_n^{IC1}$ and $PV_n^{IC2}$ respectively represent the potential of solar PV installed capacity in province $n$ by the two methods (W), $G_n^{V1}$ and $G_n^{V2}$ respectively represent the annual potential of solar PV generation in province $n$ by the two methods (Wh). The aggregated power generations are compared with the annual actual solar PV generation potential in each province, and the result with less error will be selected as an objective method for estimating this province's solar PV installed capacity and annual generation potential. The potential of installed capacity and annual potential of generation of solar PV in each province are shown in Supplementary Table 4.

**Supplementary Table 4. Potential installed capacity and annual generation potential of wind power and solar PV**

| Province | Onshore wind (GW) | Offshore wind (GW) | Solar PV (GW) | Onshore wind (TWh/a) | Offshore wind (TWh/a) | Solar PV (TWh/a) |
|---|---|---|---|---|---|---|
| Beijing | 2.78 | 0 | 19.26 | 6.20 | 0 | 31.87 |
| Tianjin | 4.37 | 1.56 | 9.94 | 14.10 | 4.91 | 15.12 |
| Hebei | 206.20 | 6.31 | 166.85 | 339.42 | 23.01 | 227.26 |
| Shanxi | 62.34 | 0 | 300.63 | 139.42 | 0 | 401.83 |
| Inner Mongolia | 2195.42 | 0 | 8913.60 | 7210.26 | 0 | 12805.87 |
| Liaoning | 57.77 | 19.21 | 166.05 | 220.49 | 86.62 | 226.76 |
| Jilin | 106.42 | 0 | 323.55 | 411.42 | 0 | 435.93 |
| Heilongjiang | 273.96 | 0 | 284.58 | 983.87 | 0 | 376.01 |
| Shanghai | 1.93 | 2.22 | 40.41 | 9.20 | 10.72 | 59.12 |
| Jiangsu | 44.25 | 12.89 | 295.32 | 133.66 | 54.03 | 378.01 |
| Zhejiang | 10.46 | 19.85 | 101.73 | 22.38 | 94.90 | 120.62 |
| Anhui | 71.34 | 0 | 229.62 | 183.24 | 0 | 308.16 |
| Fujian | 25.44 | 14.33 | 81.30 | 41.25 | 78.74 | 98.14 |
| Jiangxi | 29.88 | 0 | 98.03 | 74.47 | 0 | 130.22 |
| Shandong | 110.76 | 23.42 | 404.37 | 294.66 | 88.50 | 567.97 |
| Henan | 99.60 | 0 | 298.98 | 227.22 | 0 | 465.10 |
| Hubei | 38.80 | 0 | 163.26 | 93.38 | 0 | 209.33 |
| Hunan | 30.04 | 0 | 90.75 | 69.32 | 0 | 104.81 |
| Guangdong | 65.24 | 25.09 | 208.78 | 167.78 | 104.47 | 261.33 |
| Guangxi | 70.00 | 0.68 | 198.69 | 201.94 | 2.63 | 245.99 |
| Hainan | 13.75 | 7.50 | 25.35 | 53.89 | 22.17 | 41.89 |
| Chongqing | 6.77 | 0 | 2.79 | 15.10 | 0 | 2.51 |
| Sichuan | 285.47 | 0 | 128.05 | 294.76 | 0 | 131.44 |
| Guizhou | 33.30 | 0 | 95.97 | 96.20 | 0 | 89.76 |
| Yunnan | 59.77 | 0 | 110.70 | 174.34 | 0 | 158.96 |
| Shaanxi | 234.36 | 0 | 315.48 | 213.72 | 0 | 367.67 |
| Gansu | 472.59 | 0 | 2588.52 | 913.84 | 0 | 3401.48 |
| Qinghai | 651.42 | 0 | 3807.72 | 893.97 | 0 | 5163.98 |
| Ningxia | 64.40 | 0 | 264.12 | 172.58 | 0 | 403.91 |
| Xinjiang | 1350.22 | 0 | 20126.40 | 2581.57 | 0 | 34801.28 |
| Tibet | 1152.26 | 0 | 3317.01 | 1907.20 | 0 | 5737.58 |
| Total | 7831.29 | 133.05 | 43177.81 | 18160.84 | 570.72 | 67769.94 |

## **Supplementary Note 1.3** Firm non-fossil fuel power assessment

The installed capacity potential of hydropower and nuclear power are calculated based on monthly-adjusted 2050 projections for each province from existing literature [21] by Equations (14)-(15). Considering that both electricity supply sources are stable, we assume that they provide the same amount of electricity per hour in different months.

$$HP_{m,n}=\frac{Cap_n^{FHP}}{Cap_n^{NHP}}\cdot(Gen_{m,n}^{HP}/D_m^{all}/24) \tag{14}$$

$$NC_{m,n}=\frac{Cap_n^{FNC}}{Cap_n^{NNC}}\cdot(Gen_{m,n}^{NC}/D_m^{all}/24) \tag{15}$$

where $HP_{m,n}$. and $NC_{m,n}$ denote the hourly hydropower and nuclear power output at month *m* in province *n* in 2050 (MW), $Cap_n^{FHP}$ and $Cap_n^{FNC}$ are the installed capacity of hydropower and nuclear power in province *n* in 2050 (MW), $Cap_n^{NHP}$ and $Cap_n^{NNC}$ are the installed capacity of hydropower and nuclear power in province *n* in 2019 (MW), $Gen_{m,n}^{HP}$ and $Gen_{m,n}^{NC}$ are the power generation of hydropower and nuclear power at month *m* in province *n* in 2019 (MWh), and $D_m^{all}$ is the number of days at month *m*.

## **Supplementary Note 1.4** The share of cumulative unmet electricity demand via hourly non-fossil resource power generation potential

This indicator represents the cumulative unmet ideal electricity demand by province relative to provincial overall non-fossil fuel energy resource potential divided by the national overall ideal electricity demand, as shown in Equation (16).

$$UEDRP^N=\frac{\sum_n\left(\max\left(\sum_t IED_{n,t}-\sum_t\left(WP_{n,t}+PV_{n,t}+HP_{n,t}+NC_{n,t}\right),0\right)\right)}{\sum_n\sum_t IED_{n,t}} \tag{16}$$

where $UEDRP^N$ represents the share of cumulative unmet electricity demand via hourly non-fossil resource power generation potential, $IED_{n,t}$ is the ideal electricity demand in 2050 at hour *t* in province *n*, $WP_{n,t}$ is the wind power output potential at hour *t* in province *n*, $PV_{n,t}$ is the solar PV power output potential at hour *t* in province *n*, $HP_{n,t}$ is the hydropower output potential at hour *t* in province *n*, $NC_{n,t}$ is the nuclear power output potential at hour *t* in province *n*.

# Supplementary Note 2 CCUS source-sink matching model for fossil fuel power generation plants

In this study, the existing coal-fired and gas-fired power plants in China are used as carbon emission sources, and the onshore and offshore saline aquifers and oil fields in China are used as sequestration sinks. The assumptions of the source-sink matching model are as follows.

1. To ensure the economics of CCUS retrofitting fossil fuel power plants, this study assumes that fossil fuel power plants with installed capacities greater than 300 MW [22] and remaining lifetime longer than 15 years can be retrofitted with CCUS projects.

2. Based on actual engineering project experience and the diffusion rate of $CO_2$ in reservoirs, the area of a $CO_2$ storage grid is assumed to be 400 $km^2$ (20 km×20 km).

3. Only one injection well is assumed to be drilled in each grid.

4. A one-way source-sink matching rule is adopted, i.e., a source can only be matched to one sink, and a sink can be matched to multiple sources depending on its storage potential and injection rate capacity.

5. Geological reservoirs associated with deep saline aquifers and oil fields are considered as candidate storage sites; note that here the oil fields are assumed to be identified as a purposely operated $CO_2$ sequestration option with matching priority due to the benefits of enhanced oil recovery (EOR).

6. Due to the relatively high cost of offshore storage [23], onshore storage is preferred in the source-sink matching process. Therefore, matching storage sites are prioritized as: onshore oil field > offshore oil field > onshore saline aquifer > offshore saline aquifer.

7. Regarding the sequence of matched emission sources, fossil power plants with higher $CO_2$ emissions are prioritized in the source-sink matching process to maximize the amount of $CO_2$ storage.

8. Considering that a very high $CO_2$ injection rate will lead to the destruction of geological layers, thus causing $CO_2$ leakage and earthquakes [24], the model incorporates a constraint of $CO_2$ injection rate capacity in addition to $CO_2$ storage potential.

9. Considering the economics of $CO_2$ transport, the upper limit for $CO_2$ transport distance from fossil power plants to storage sites is assumed to be 500 km.

The CCUS source-sink matching model was solved using Python and ArcGIS software, where the objective functions are to maximize $CO_2$ storage and minimize $CO_2$ transport equivalent, as shown in Equations (17).

$$\begin{cases} \max TS = \sum_{p} \sum_{s \in S_x} \left( X_{p,s} \cdot S_{p,s} \right) \\ \min TT = \sum_{p} \sum_{s \in S_x} \left( X_{p,s} \cdot D_{p,s} \cdot S_{p,s} \right) \end{cases} \tag{17}$$

where $TS$ and $TT$ denote total $CO_2$ storage (t) and total $CO_2$ transport equivalent (t·km) attributed to all possible source-sink matching results. $S_x$ is four various sequestration options, where $S_1$ is the set of onshore oil field, $S_2$ is the set of offshore oil field, $S_3$ is the set of onshore saline aquifer, $S_4$ is the set of offshore saline aquifer. $X_{p,s}$ is a binary variable with $X_{p,s} = 1$ indicating power plant *p* and storage site *s* are matched successfully, otherwise, $X_{p,s} = 0$. $S_{p,s}$ is the annual $CO_2$ storage amount of power plant *p* when power plant *p* and storage site *s* are matched successfully,

and $D_{p,s}$ is the transport distance from power plant *p* and storage site *s*.

The constraints of the source-sink matching model are primarily as follows: first, the cumulative $CO_2$ sequestration of each storage site does not exceed its maximum storage potential; second, the actual source-sink matching distance of each plant does not exceed the maximum distance limit; and third, the annual $CO_2$ injection of each storage site does not exceed its maximum injection rate capacity. The constraints are shown in Equation (18).

$$\begin{cases} \sum_p X_{p,s} \cdot S_{p,s} \le SP_s, & \forall s \in [1, S_x] \\ 0 \le X_{p,s} \cdot D_{p,s} \le D^*, & \forall p \in [1, N], s \in [1, S_x] \\ \sum_p X_{p,s} \cdot S_{p,s} / Y_p \le I_s, & \forall s \in [1, S_x] \end{cases} \tag{18}$$

where $SP_s$ is the $CO_2$ storage potential of sink *s* (t). $D^*$ is the maximum transport distance of 500km. $Y_p$ is the operational lifetime of plant *p* (a), assuming that the storage site needs to meet $CO_2$ emissions from the source over its 30-year operation cycle, and $I_s$ is the annual injection rate capacity of sink *s* (t/a).

# Supplementary Note 3 Modules for the optimal near-zero power system simulation model

Considering the general equilibrium-based top-down model may distort the costs of intermittent renewable power when modelling the power system, which is limited by the model assumption of a constant elasticity of substitution production function[25], the cost-optimized bottom-up model based on linear programming is more appropriate for the scientific questions in this study. However, an hourly cost-optimized power system model that takes into account inter-provincial power transmission is very time consuming to solve. To make the model can be solved in sensible computational time, some of the cost-optimized power system models consider a country as a whole and do not consider regional and inter-provincial power transmission to simplify the model, which may lead to bias in the results. Another clustering-based cost-optimized intertemporal power system model that consider power transmission generally suffers from omitting fluctuation details of variable renewable resource potential [26], clustering bias of mixing electricity consumption and renewable energy generation potential [27], and inability to accurately assess power shortages especially under extreme climatic events [28, 29], e.g., clustering results are difficult to capture the near real-time changes in meteorological factors at a specific hour during a climatic disaster, resulting in power shortages that cannot be accurately assessed. Therefore, considering the limitations of the above models and aiming at a simultaneous assessment of the power system's reliability and resilience, this study adopts an iterative-based approach to construct an inter-provincial power system simulation model based on near real-time hourly meteorological data and calculate the hourly power shortages during normal year and the period of extreme climatic events (snowstorms, sandstorms, heat waves, and droughts), respectively.

## **Supplementary Note 3.1** Inclusion mechanism of Long-term energy storage in the near-zero power system simulation model

Considering the future application prospects hydrogen energy storage [30], we adopt hydrogen energy storage as the form of long-term energy storage in this study. Following previous prediction of hydrogen consumption [31], we assume 15% of the end-use energy demand in 2050 (90 EJ [32]) from hydrogen, 70% of which is provided by electrolytic water [31]. The hydrogen production is tended to boost the electricity demand of each province by the same amplification factor. Since the hydrogen energy can be utilized via various ways, it is assumed up to 5% of the national hydrogen energy can be used in the electricity system as long-term electricity storage [33], and they are allocated to different provinces according to the degrees of power shortage in each province. Like the short-term energy storage, local long-term energy storage discharging and hourly dispatch via long-term energy storage discharging are included in the electricity supply system, with prioritizing to local long-term energy storage discharging.

## **Supplementary Note 3.2** Calculated amount of electricity supply from various sources

Based on the constraints of the optimal near-zero power system simulation model, the local real-time hourly electricity supply via power generation, real-time hourly dispatch electricity supply via power generation, local energy storage discharging electricity supply, and electricity dispatch

via energy storage discharging electricity supply can be calculated by the following Equations (19) -(22).

$$ES_{n,t}^{l} = \min\left(IED_{n,t}, \sum_{z} PGP_{z,n,t}\right) \tag{19}$$

where $ES_{n,t}^{l}$ is local the real-time hourly electricity supply via power generation at hour $t$ in province $n$, as shown in Equation (15) in Method section. $IED_{n,t}$ is the ideal electricity demand in 2050 at hour $t$ in province $n$, as shown in Equation (14) in Method section. $PGP_{z,n,t}$ is the power generation potential of the various power generation technologies $z$ at hour $t$ in province $n$, where $z=1$ is onshore wind power, $z=2$ is offshore wind power, $z=3$ is solar PV power, $z=4$ is nuclear power, $z=5$ is hydropower, $z=6$ is coal-fired power with CCUS, and $z=7$ is natural gas-fired power with CCUS, as shown in Equation (18) in Method section.

$$ES_{n,t}^{d} = \min\begin{pmatrix} IED_{n,t} - ES_{n,t}^{l}, \sum_{n'} X_{n',n} \cdot DC_{n',n}, \\ \sum_{n'} X_{n',n} \cdot \left(\sum_{z} PGP_{z,n',t} - IED_{n',t} - EDS_{n',n,t}^{d}\right) \end{pmatrix} \tag{20}$$

where $ES_{n,t}^{d}$ is the real-time hourly dispatch electricity supply via power generation from other provinces at hour $t$ in province $n$, as shown in Equation (15) in Method section. $X_{n',n}$ is a binary variable and is assigned a value of 1 if there is a transmission line from dispatched outflow province $n'$ to dispatched inflow province $n$. Otherwise, a value of 0 is assigned, as shown in Equation (20) in Method section. $DC_{n',n}$ is the maximum transmission capacity from dispatched outflow province $n'$ to dispatched inflow province $n$, as shown in Equation (20) in Method section. $PGP_{z,n',t}$ is the power generation potential of the various power generation technologies $z$ at hour $t$ in province $n'$, as shown in Equation (21) in Method section. $IED_{n',t}$ is the ideal electricity demand in 2050 at hour $t$ in province $n'$, as shown in Equation (21) in Method section. $EDS_{n',n,t}^{d}$ is the accumulation of electricity dispatch via power generation from province $n'$ to the other provinces prioritized over province $n$ at hour $t$ (if province $n$ is the most electricity-deficient province and all other provinces are prioritized to supply electricity to province $n$, $EDS_{n',n,t}^{d}$ is 0), as shown in Equation (21) in Method section.

$$ES_{n,t}^{s} = \min\left(IED_{n,t} - ES_{n,t}^{l} - ES_{n,t}^{d}, \sum_{h=t-h0-H+1}^{t-h0}\left(RWP_{n,h} + RPV_{n,h}\right) - \sum_{h=t-h0+1}^{t-1}\left(ES_{n,h}^{s} + EDS_{n,h}^{sd}\right)\right) \tag{21}$$

where $ES_{n,t}^{s}$ is the real-time hourly electricity supply comes from energy storage discharging at hour $t$ in province $n$, as shown in Equation (15) in Method section. $h$ is an auxiliary variable related to $t$ for simulating the process of energy storage charging and discharging, as shown in Equation (23) in Method section. $h0$ is the number of hours from the end of energy storage charging to hour $t$, as shown in Equation (23) in Method section. $H$ is the maximum number of energy storage hours, which ranges from 1 to 24 hours according to the different scenarios, as shown in Equation (23) in Method section. $RWP_{n,h}$ is the remaining power generation potential of wind power after the local real-time hourly electricity supply and real-time hourly dispatch electricity supply at hour $h$ in province $n$, as shown in Equation (23) in Method section. $RPV_{n,h}$ is the remaining power generation

potential of solar PV power after the local real-time hourly electricity supply and real-time hourly dispatch electricity supply at hour $h$ in province $n$, as shown in Equation (23) in Method section. $ES^{s}_{n,h}$ is the local energy storage discharging electricity supply at hour $h$ in province $n$, as shown in Equation (23) in Method section. $EDS^{sd}_{n,h}$ is the electricity dispatch via energy storage discharging from province $n$ at hour $h$, as shown in Equation (23) in Method section.

$$ES^{sd}_{n,t} = \min\left( \begin{array}{l} IED_{n,t} - ES^{l}_{n,t} - ES^{d}_{n,t} - ES^{s}_{n,t}, \sum_{n'} X_{n',n} \cdot \left( DC_{n',n} - ES^{d}_{n',n,t} \right), \\ \sum_{n'} X_{n',n} \cdot \left( \begin{array}{l} \sum_{h=t-h0-H+1}^{t-h0} \left( RWP_{n',h} + RPV_{n',h} \right) - \\ \sum_{h=t-h0+1}^{t-1} (ES^{s}_{n',h} + EDS^{sd}_{n',h}) - \left( ES^{s}_{n',t} + EDS^{sd\_o}_{n',n,t} \right) \end{array} \right) \end{array} \right) \tag{22}$$

where $ES^{sd}_{n,t}$ is the hourly electricity supply from other provinces to province $n$ at hour $t$ through hourly dispatch via energy storage discharging, as shown in Equation (15) in Method section. $ES^{d}_{n',n,t}$ is the real-time hourly dispatch electricity supply via power generation from province $n'$ to province $n$ at hour $t$, as shown in Equation (25) in Method section. $RWP_{n',h}$ is the remaining power generation potential of wind power after the local real-time hourly electricity supply and real-time hourly dispatch electricity supply at hour $h$ in province $n'$, as shown in Equation (26) in Method section. $RPV_{n',h}$ is the remaining power generation potential of solar PV after the local real-time hourly electricity supply and real-time hourly dispatch electricity supply at hour $h$ in province $n'$, as shown in Equation (26) in Method section. $ES^{s}_{n',h}$ is the local energy storage discharging electricity supply at hour $h$ in province $n'$, as shown in Equation (26) in Method section. $EDS^{sd}_{n',h}$ is the electricity dispatch via energy storage discharging from province $n'$ at hour $h$, as shown in Equation (26) in Method section. $ES^{s}_{n',t}$ is the local energy storage discharging electricity supply at hour $t$ in province $n'$, as shown in Equation (26) in Method section. $EDS^{sd\_o}_{n',n,t}$ is the accumulated electricity dispatch via energy storage discharging dispatched from province $n'$ to province $n$ at hour $t$, as shown in Equation (26) in Method section.

# Supplementary Note 4 Cost calculation for power generation technologies

The approach for assessing power system costs, including non-fossil power generation costs, abated fossil fuel power generation costs, energy storage costs, and electricity transmission costs, is introduced in this note.

## **Supplementary Note 4.1** The cost of non-fossil fuel power generation

The Levelized Cost of Energy (LCOE) of non-fossil fuel power generation is calculated considering technological progress, as shown in Equation (23).

$$LCOE_k = LCOE_k' \cdot \left(1 - CR_k\right) \tag{23}$$

where $LCOE_k$ is the LCOE of zero fossil fuel power generation technologies *k* in 2050 (at 2020 constant price) (USD/kWh), where $k=1$ is onshore wind power, $k=2$ is offshore wind power, $k=3$ is solar PV power, $k=4$ is nuclear power, and $k=5$ is hydropower. $LCOE_k'$ is the LCOE of zero fossil fuel power generation technologies *k* in 2020 (USD/kWh), respectively. $CR_k$ is the LCOE reduction rate of power generation technologies *k* in 2050. The detailed values and sources can be found in Supplementary Table 5.

## **Supplementary Note 4.2** The cost of abated fossil fuel power generation with CCUS

Coal-fired and gas-fired power plants are assumed to operate continuously after the CCUS retrofit. As existing coal-fired and gas-fired power plants are considered as CCUS retrofitting candidate in the power system, the construction cost of these plants is not included into the LCOE calculation. The LCOE of coal-fired power and nature gas-fired power generation with CCUS are calculated by Equation (24).

$$LCOE_{k'}^{fl\_ccs} = \frac{\sum_p \left( O\&M_{k'} \cdot Cap_p + h_p \cdot Cap_p \cdot \sigma_{k'} \cdot P_{k'} + (CCS^c + CCS^t \cdot L_{k'} + CCS^s) \cdot CER_p \right)}{\sum_p Q_p} \tag{24}$$

where $LCOE_{k'}^{fl\_ccs}$ is the LCOE of fossil fuel power generation with CCUS (USD/kWh), where $k'=1$ is coal-fired power generation with CCUS, $k'=2$ is nature gas-fired power generation with CCUS. $O\&M_{k'}$ is the annual operation and maintenance cost of fossil fuel power generation plants (USD/kW). $Cap_p$ is the installed capacity of fossil fuel power plant *p* (kW). $h_p$ is the annual operation time of fossil fuel power plant *p* (h). $\sigma_{k'}$ is the fossil fuel consumed of fossil fuel power generation plant (t/kWh or m³/ kWh). $P_{k'}$ is the fossil fuel price (USD/t or USD/m³). $CCS^c$ is the unit $CO_2$ capture cost in 2050 (USD/t). $CCS^t$ is the unit $CO_2$ transport cost in 2050 (USD/t·km). $L_{k'}$ is the weighted average source-sink distance at the current fossil fuel ratio (km), and is taken from the result of the source-sink matching model. $CCS^s$ is the unit $CO_2$ storage cost in 2050 (USD/t). $CER_p$ is the annual $CO_2$ capture from fossil fuel power plant *p* (t), and $Q_p$ is the annual electricity generation of fossil fuel power plant *p* (kWh). The detailed values can be found in Supplementary Table 5.

## **Supplementary Note 4.3** The cost of energy storage

Based on the technical maturity, development prospect and experts survey of each energy storage technology, we adopt Li-ion battery energy storage to represent short-term energy storage technology and hydrogen energy storage to represent long-term energy storage in the calculation of power system cost. The LCOE of energy storage (short-term and long-term) is calculated considering technological progress, as shown in Equations (25)-(26).

$$LCOE^{es} = LCOE^{es'} \cdot \left(1 - CR^{es}\right) \tag{25}$$

$$H2^{s} = H2^{s'} \cdot \left(1 - CR^{H2S}\right) \tag{26}$$

where $LCOE^{es}$ is the LCOE of short-term energy storage in 2050 (at 2020 constant price) (USD/kWh). $LCOE^{es'}$ is the LCOE of short-term energy storage in 2020 (USD/kWh). $CR^{es}$ is the LCOE reduction rate of short-term energy storage in 2050. $H2^{s}$ is the cost of hydrogen storage in 2050 (at 2020 constant price) (USD/kg). $H2^{s'}$ is the cost of hydrogen storage in 2015 (at 2020 constant price) (USD/kg). $CR^{H2S}$ is the decrease rate in hydrogen cost from 2015 to 2050, taken as 0.175. In addition, the cost of hydrogen production from renewable energy can be referred to Equation (45) in the Method section, and as for hydrogen combustion power generation, we only consider the operation and maintenance cost, assuming that it can use the infrastructure of existing fossil fuel power plants. The detailed values and sources can be found in Supplementary Table 5.

## **Supplementary Note 4.4** The cost of power transmission lines

Assuming that the construction period of Ultra High Voltage (UHV) lines is normally 2 years and the new UHV lines will be completed in 2030 with an operational life of 30 years [34], the unit transmission cost is calculated by Equations (27)-(28).

$$COST^{UT} = ACOST^{UT} / L^{d} / Cap^{t} \tag{27}$$

where $COST^{UT}$ is the unit transmission cost (at 2020 constant price) (USD/(km·GW)). $ACOST^{UT}$ is the annual average cost of the selected UHV line (USD), which can be calculated by Equation (28). $L^{d}$ is the length of the specific UHV line (km), and $Cap^{t}$ is the transmission capacity of the specific UHV line (GW). The detailed values and sources can be found in Supplementary Table 5.

$$ACOST^{UT} = \frac{D^{cc} \cdot e^{-\alpha \cdot t^{s}} + D^{OM} \cdot \dfrac{e^{-r_0} - e^{r_0 \cdot (\tau_1 - \tau_2)}}{e^{r_0} - 1}}{\dfrac{e^{-r_0} - e^{r_0 \cdot (\tau_1 - \tau_2)}}{e^{r_0} - 1}} \tag{28}$$

where $D^{cc}$ denotes the construction cost of UHV lines (USD), which is referred to the actual cost of Ningxia-Zhejiang UHV line. $D^{cc'}$ denotes the reference construction cost of the actual Ningxia-Zhejiang UHV line in 2014, assuming it is unchanged if measured by 2020 price. $\alpha$ is the learning rate of UHV lines, taken as 5% [35]. $t^{s}$ is the study time of UHV lines, taken as 15 years (2014-2028). $D^{OM}$ is the annual operation and maintenance cost of the UHV lines (USD). $r_0$ is the discount rate, taken as 5%. $\tau_1$ and $\tau_2$ represent the initial and end of operation years of UHV respectively. The detailed values and sources can be found in Supplementary Table 5.

**Supplementary Table 5. Determination of key cost parameters**

| Parameters | Value | Data sources |
|---|---|---|
| $O\&M^{cp}$ (USD/kW) | 19.28 | Reference [36] |
| $\sigma^{cp}$ (g/kWh) | 302.5 | Reference [37] |
| $P^{coal}$ (USD/t) | 74.30 | Average Coal Prices in China by Province 2018-2019 [38] |
| $CCS^{c}$ (USD/t) | 16.67 | Average unit capture cost in 2050 [23] |
| $CCS^{t}$ (USD/t·km) | 0.065 | Average unit transportation cost in 2050 [23] |
| $CCS^{s}$ (USD/t) | 3.99 | Average unit sequestration cost in 2050 [23] |
| $O\&M^{ng}$ (USD/kW) | 15.37 | Reference [36] |
| $\sigma^{ng}$ ($m^3$/ kWh) | 0.2 | Reference [36] |
| $P^{ng}$ (USD/$m^3$) | 0.25 | Average natural gas prices by province [39] |
| $D^{cc'}$ (B USD) | 3.44 | Reference [40] |
| $D^{OM}$ (B USD) | 0.057 | Reference [34, 40] |
| $L^{d}$ (km) | 1720 | Reference [40] |
| $Cap^{t}$ (GW) | 8 | Reference [40] |
| $LCOE^{nc'}$ (USD/kWh) | 0.066 | Reference [41] |
| $LCOE^{hp'}$ (USD/kWh) | 0.043 | Reference [42] |
| $LCOE^{on-wp'}$ (USD/kWh) | 0.058 | Reference [41] |
| $LCOE^{off-wp'}$ (USD/kWh) | 0.082 | Reference [41] |
| $LCOE^{pv'}$ (USD/kWh) | 0.051 | Reference [41] |
| $LCOE^{es'}$ (USD/kWh) | 0.097 | Reference [4] |
| $H2^{uc}$ (USD/kg) | 1.2 | Reference [43] |
| $PEC^{H2}$ | 0.7 | Reference [31] |
| $H2^{s}$ (USD/kg) | 0.45 | Reference [44] |
| $CR^{H2S}$ | 0.005 | Reference [45] |
| $CR^{nc}$ | 0.00889 | Refer to 2050 compared to the current cost reduction [46] |
| $CR^{hp}$ | 0.165 | Refer to Japan 2050 compared to the current cost reduction [47] |
| $CR^{on-wp}$ | 0.37 | Cost reduction in 2050 compared to current costs under the medium scenario [48] |
| $CR^{off-wp}$ | 0.47 | Cost reduction in 2050 compared to current costs under the medium scenario [48] |
| $CR^{pv}$ | 0.37 | Cost reduction in 2050 compared to current costs under the medium scenario [49] |
| $CR^{es}$ | 0.57 | Refer to 2050 compared to the current cost reduction [50] |

Note: For the cost reduction of onshore wind power, offshore wind power, and solar PV, we used the medium scenario of all cost reduction scenarios in the corresponding references [48, 49].

# Supplementary Note 5 Impact mechanism of extreme weather events on the power system

The mechanisms and specific simulation methods for the impact of different extreme climate disasters (i.e., snowstorms, sandstorms, droughts, and heat waves) on the power system is introduced in this note.

## **Supplementary Note 5.1** Impact mechanism of snowstorms

Snowstorms often have three effects on the near-zero power system. First, continuous snowfall can cause snow deposition on solar PV panels, drastically reducing solar PV output efficiency [51]. The specific process by which snowstorms affect solar PV can be expressed by Equation (29).

$$PV_{n,t}^{sn'} = \min\left(PV_{n,t}^{sn}, PV_{n}^{IC}\right) \cdot EPV_{n,t}^{sn} \tag{29}$$

where $PV_{n,t}^{sn'}$ is the actual solar PV power output at hour $t$ in province $n$ during the snowstorm. $PV_{n,t}^{sn}$ is the solar PV power output potential at hour $t$ in province $n$ during the snowstorm. $PV_{n}^{IC}$ is the installed capacity of solar PV of province $n$ under the optimal scenario in order to make the results of both scenarios comparable (so do other events and other non-fossil power generation). $EPV_{n,t}^{sn}$ is the change coefficient of output efficiency of solar PV power generation at hour $t$ in province $n$ relative to the absence of snow, and is related to snowfall thickness during the snowstorm, the values can be found in Supplementary Table 6.

**Supplementary Table 6. Relationship between snow thickness and transmittance** [52]

| Snow depth（cm） | 0 | 2 | 4 | 6 | 8 | >10 |
|---|---|---|---|---|---|---|
| Transmittance（%） | 1 | 0.175 | 0.1 | 0.076 | 0.058 | 0 |

Note: Referring to the relevant literature, the medium wavelength of light (650nm) in sunlight is chosen. The snowfall thickness of the PV panel is calculated according to the actual snow depth on the ground after considering the inclination angle of the PV panel.

Second, snowstorms make wind turbines in affected areas prone to freezing, drastically reducing wind power output efficiency [53]. The specific process by which snowstorms affect wind power can be expressed by Equation (30).

$$WP_{n,t}^{sn'} = \min\left(WP_{n,t}^{sn}, WP_{n}^{IC}\right) \cdot EWP_{n,t}^{sn} \tag{30}$$

where $WP_{n,t}^{sn'}$ is the actual wind power output at hour $t$ in province $n$ during the snowstorm, $WP_{n,t}^{sn}$ is the wind power output potential at hour $t$ in province $n$ during the snowstorm, $WP_{n}^{IC}$ is the installed capacity of wind power of province $n$ under the optimal scenario, and $EWP_{n,t}^{sn}$ is the change coefficient of output efficiency of wind power generation at hour $t$ in province $n$ during the snowstorm relative to normal weather, as shown in Equation (31) [54].

$$EWP_{n,t}^{sn} = 100\% - (2.119 \cdot h_{n,t}^{ice} + 1.553)\% \tag{31}$$

where $h_{n,t}^{ice}$ is the ice thickness at hour $t$ in province $n$ during the snowstorm (mm). The relationship between snowfall and freezing thickness can be simulated using the freezing equation [55]. Specifically, assuming that the contact area between the snow and the wind turbine is half the area of the wind turbine, the freezing equation can be simplified as shown in Equations (32)-(33).

$$h_{n,t}^{ice} = \frac{\alpha_1 \cdot \alpha_2 \cdot \alpha_3 \cdot w_{n,t} \cdot d_{n.,t}^{sn} \cdot 2}{\rho^{ice} \cdot 1000} \tag{32}$$

$$\begin{cases} \alpha_2 = \dfrac{1}{v_{n,t}}, v_{n,t}^{sn} \geq 1 \\ \alpha_2 = 1, v_{n,t}^{sn} \leq 1 \end{cases} \tag{33}$$

where $\alpha_1$ is the collision coefficient (1 for wet snow) [56]. $\alpha_2$ is the viscosity coefficient. $\alpha_3$ is the accretion coefficient (1 for wet snow) [56]. $w_{n,t}$ is the mass concentration of snow particles at hour $t$ in province $n$ during the snowstorm (kg/m$^3$). $d_{n.,t}^{sn}$ is the distance that the snow particles move per second at hour $t$ in province $n$ (m). $\rho^{ice}$ is the ice density, taken as 917kg/m$^3$. $v_{n,t}$ is the wind speed at hour $t$ in province $n$ during the snowstorm (m/s), and $v_{n,t}^{sn}$ is the snow particle velocity at hour $t$ in province $n$ during the snowstorm (m/s).

Third, the snowstorm will seriously damage power transmission infrastructures and reduce their transmission capacities. Although direct-current (DC) melting devices have been widely installed to power grid after 2008 snowstorms, the destruction of snowstorms on power transmission still cannot be ignored due to the limited application conditions of DC melting devices [57], the complex and various causes for transmission line damage [58], and the likely enhanced frequency and magnitude of extreme weather in the future [59]. Combined with the actual loss of transmission lines over 220 kV in Jiangxi, Guizhou, and Hunan during the 2008 snowstorm [60], up to 60.5% of inter-provincial lines related to the affected provinces were assumed to be damaged.

## **Supplementary Note 5.2** Impact mechanism of sandstorms

Sandstorms often have two effects on the near-zero power system. First, sandstorms cause a significant reduction in radiation intensity, resulting in lower solar PV output efficiency; in addition, dust deposition on solar PV panels further reduce solar PV output efficiency [61]. The specific process by which sandstorms affect solar PV can be expressed by Equation (34).

$$PV_{n,t}^{sa'} = \min\left(PV_{n,t}^{sa}, PV_{n}^{IC}\right) \cdot EPV_{n,t}^{sa} \cdot R_{n,t}^{sa} \tag{34}$$

where $PV_{n,t}^{sa'}$ is the actual solar PV power output at hour $t$ in province $n$ during the sandstorm. $PV_{n,t}^{sa}$ is the solar PV power output potential at hour $t$ in province $n$ during the sandstorm. $EPV_{n,t}^{sa}$ is the change coefficient of output efficiency of solar PV power generation at hour $t$ in province $n$ during the sandstorm relative to normal weather, and $R_{n,t}^{sa}$ is the proportion of remaining radiation intensity at hour $t$ in province $n$ during the sandstorm, the values can be found in Supplementary Table 7.

Second, sand and gravel can wear down turbine impellers; in severe cases, resulting in a reduction in wind turbine output efficiency [61]. The specific process by which sandstorms affect wind power can be expressed by Equation (35).

$$WP_{n,t}^{sa'} = \min\left(WP_{n,t}^{sa}, WP_{n}^{IC}\right) \cdot EWP_{n,t}^{sa} \tag{35}$$

where $WP_{n,t}^{sa'}$ is the actual wind power output at hour *t* in province *n* during the sandstorm. $WP_{n,t}^{sa}$ is the wind power output potential at hour *t* in province *n* during the sandstorm, and $EWP_{n,t}^{sa}$ is the change coefficient of output efficiency of wind power generation at hour *t* in province *n* during the sandstorm relative to normal weather, the values can be found in Supplementary Table 7, and the starting and ending times of the provinces affected by sandstorms are shown in Supplementary Table 8.

**Supplementary Table 7. The effect of sandstorms on wind power and solar PV output efficiency**

| Disaster intensity | Solar PV efficiency[62] | Radiation intensity[63] | Wind power efficiency[64] |
|---|---|---|---|
| Floating and sinking or sand blowing | 0.88 | 0.2187 | 0.85 |
| Sandstorm | 0.8415 | 0.1294 | 0.70 |
| Severe sandstorm | 0.7372 | 0.0647 | 0.40 |

Note: According to the classification of sandstorms, the particle concentration and the visibility range of the strong sandstorm is about twice and a half that of a typical sandstorm, respectively [65]. Therefore, the radiation intensity during a strong sandstorm in this study is taken as a half of the intensity during a sandstorm. In addition, since the output efficiency loss of wind power is linearly related to the thickness of dust accumulation in wind turbine [66], the output efficiency loss of wind power during a strong sandstorm is taken as twice that during a typical sandstorm.

**Supplementary Table 8. Sandstorm duration in affected areas**

| Province | Start time | End time |
|---|---|---|
| Western Inner Mongolia | March 14, 11:00 | March 16, 20:00 |
| Xinjiang | March 14, 22:00 | March 16, 24:00 |
| Gansu | March 14, 22:00 | March 19, 24:00 |
| Ningxia | March 15, 2:00 | March 19, 24:00 |
| Beijing | March 15, 6:00 | March 16, 8:00 |
| Tianjin | March 15, 6:00 | March 16, 8:00 |
| Shanxi | March 15, 6:00 | March 16, 24:00 |
| Hebei | March 15, 7:00 | March 16, 24:00 |

Note: Here the affected areas of the sandstorm only include the seriously affected provinces [67], and the affected time is referred to the news of the occurrence of the disaster [68, 69, 70, 71, 72, 73, 74].

## **Supplementary Note 5.3** Impact mechanism of droughts

Droughts often have three effects on the near-zero power system. First, droughts cause significant evaporation of water, resulting in insufficient reservoir storage and reductions in hydropower output. The specific process by which droughts affect hydropower can be expressed by Equation (36).

$$HP_{n,t}^{dr'} = HP_{n,t}^{dr} \cdot EHP_{n,t}^{dr} \tag{36}$$

where $HP_{n,t}^{dr'}$ is the actual hydropower output at hour *t* in province *n* during the drought. $HP_{n,t}^{dr}$ is the power output potential at hour *t* in province *n* during the drought, and $EHP_{n,t}^{dr}$ is the change coefficient of output efficiency of hydropower generation at hour *t* in province *n* during the drought relative to normal weather, the values can be found in Supplementary Table 9.

Second, droughts are typically accompanied by hot weather; high temperatures during drought periods can raise societal demand for refrigeration (e.g., air conditioners), thus increasing the electricity demand. The specific process by which droughts affect electricity demand can be expressed by Equation (37).

$$ED_{n,t}^{dr} = IED_{n,t} \cdot \left(1 + \Delta T_{n,t}^{dr} \cdot I^{ED}\right) \tag{37}$$

where $ED_{n,t}^{dr}$ is the hourly electricity demand at hour *t* in province *n* during the drought. $\Delta T_{n,t}^{dr}$ is the temperature increment at hour *t* in province *n* during the drought compared to the year selected in the study (2016), and $I^{ED}$ is the percentage increase in electricity demand per 1°C higher temperature rise, taken as 2.3% [75].

Third, the high temperature of PV panels caused by hot weather reduces solar PV power output efficiency [76], as shown in Equation (S5). The solar PV and wind power output during drought periods is calculated by Equations (38)-(39).

$$PV_{n,t}^{dr'} = \min\left(PV_{n,t}^{dr}, PV_{n}^{IC}\right) \tag{38}$$

$$WP_{n,t}^{dr'} = \min\left(WP_{n,t}^{dr}, WP_{n}^{IC}\right) \tag{39}$$

where $PV_{n,t}^{dr'}$ and $WP_{n,t}^{dr'}$ are the actual solar PV and wind power output at hour *t* in province *n* during the drought respectively. $PV_{n,t}^{dr}$ and $WP_{n,t}^{dr}$ are the power output potential at hour *t* in province *n* at the corresponding radiation intensity and wind speed during the drought respectively. The impacts of droughts on hydropower are shown in Supplementary Table 9, and the starting and ending times of the provinces affected by droughts are shown in Supplementary Table 10.

**Supplementary Table 9. The effect of droughts on hydropower output efficiency**

| Disaster intensity | Hydropower remaining proportion |
|---|---|
| Slight (SL) | 0.80 |
| Moderate (MO) | 0.60 |
| Severe (SE) | 0.50 |
| Extremely severe (ES) | 0.40 |

Note: In 2022, Sichuan Province experienced a 50% loss in hydropower during severe drought periods. Then we determine the remaining proportion of hydropower based on different levels of drought intensity[77].

**Supplementary Table 10. Drought duration and intensity in affected areas [78]**

| Date | Province | | | | | |
|---|---|---|---|---|---|---|
| | Sichuan | Chongqing | Hubei | Hunan | Jiangxi | Anhui |
| August 12 | MO | MO | SL | MO | MO | MO |
| August 13 | MO | MO | MO | MO | MO | MO |
| August 14 | SE | MO | SE | MO | SE | MO |
| August 15 | SE | SE | SE | MO | SE | MO |
| August 16 | SE | SE | SE | MO | SE | MO |
| August 17 | SE | SE | SE | SE | SE | MO |
| August 18 | SE | SE | SE | SE | SE | MO |
| August 19 | SE | SE | SE | SE | SE | MO |
| August 20 | SE | SE | SE | SE | SE | MO |
| August 21 | ES | SE | SE | SE | SE | MO |
| August 22 | ES | ES | SE | SE | ES | MO |
| August 23 | ES | ES | ES | SE | ES | SE |
| August 24 | ES | ES | ES | SE | ES | SE |
| August 25 | ES | ES | ES | ES | ES | ES |
| August 26 | ES | ES | ES | ES | ES | ES |
| August 27 | ES | ES | ES | ES | ES | SE |

Note: Here the affected areas of the drought only include the seriously affected provinces, and the affected time is referred to the news of the occurrence of the disaster. The intensity of drought includes four levels: Slight (SL), Moderate (MO), Severe (SE) and Extremely severe (ES).

### **Supplementary Note 5.4** Impact mechanism of heat waves

Heat waves often have three effects on the near-zero power system. First, heat waves have similar effects from the hot weather of droughts, i.e., high temperatures during heat waves can raise societal electricity demand, e.g., refrigeration equipment. The specific process by which heat waves affect electricity demand can be expressed by Equation (40).

$$ED_{n,t}^{hw} = ED_{n,t} \cdot \left(1 + \Delta T_{n,t}^{hw} \cdot I^{ED}\right) \tag{40}$$

where $ED_{n,t}^{hw}$ is the hourly electricity demand at hour $t$ in province $n$ during the heat wave, and $\Delta T_{n,t}^{hw}$ is the temperature increment at hour $t$ in province $n$ during the heat wave compared to the year selected in the study.

Second, high temperatures cause photovoltaic panels to overheat, resulting in lower solar PV output efficiency [76]. The specific process by which heat waves affect solar PV can be expressed by Equation (41).

$$PV_{n,t}^{hw'} = \min\left(PV_{n,t}^{hw}, PV_{n}^{IC}\right) \tag{41}$$

where $PV_{n,t}^{hw'}$ is the actual solar PV power output at hour $t$ in province $n$ during the heat wave, and $PV_{n,t}^{hw}$ is the solar PV power output potential at hour $t$ in province $n$ at the corresponding photovoltaic panels temperature during the heat wave.

Third, heat waves also weaken wind speeds and associated wind power output. The specific process by which heat waves affect wind power can be expressed by Equation (42).

$$WP_{n,t}^{hw'} = \min\left(WP_{n,t}^{hw}, WP_{n}^{IC}\right) \tag{42}$$

where $WP_{n,t}^{hw'}$ is the actual wind power output during the heat wave, and $WP_{n,t}^{hw}$ is the power output potential of the province at the corresponding wind speed during the heat wave.

# Supplementary Note 6 Steady state consideration of the power system

According to the electricity supply business rules issued by the Chinese National Energy Administration, the frequency rating of China's power grid is around 50 Hz [79], and the permitted frequency variation should not exceed 1.0 Hz under abnormal conditions. Thus, we assume that the power system is steady state when the national real-time hourly power shortage rate is less than 2% (i.e., 1/50).

## **Supplementary Note 6.1** Steady state of optimal power system under normal weather

How the power system can be restored to a steady state under various power shortage rate over 2% is shown in Supplementary Figure 14. In terms of observability of Lyapunov stability, 54 of 8760 hours own the national real-time hourly power shortage rate larger than 2%, with 2-3% for 36 hours, 3-4% for 11 hours, 4-5% for 6 hours, and more than 5% for 1 hour, and with 0, 32, 4, and 18 hours of national hourly power shortage rate greater than 2% in spring, summer, autumn, and winter, respectively (Supplementary Figure 14). In terms of controllability of Lyapunov stability, the fluctuation period for power shortage rate more than 2% is generally between 6 pm and 7 am. The power system can be restored to a steady state in a maximum of 7 hours when the fluctuation period is from 6 pm to 11 pm, whereas it can be restored in a maximum of 5 hours when the fluctuation period is from 0 am to 6 am (Supplementary Figure 14). This indicates that under normal weather conditions, the power output variation has little and short impacts on the electricity supply, which enables the optimal power system easily to return to a stable state.

## **Supplementary Note 6.2** Steady state of optimal power system under weather events

The power shortage rates during extreme weather events (including snowstorms, sandstorms, droughts, and heat waves) are shown in Figure 4 and Supplementary Figure 9; and the results indicate that extreme weather events have a large impact on the power system stability, mostly in the form of higher power shortage rate than the threshold value of 2%. In terms of observability of Lyapunov stability, there are 466, 17, 36, and 0 hours (101, 3, 5, and 0 hours) of power shortage rate higher than 2% (10%) in the 16% abated fossil fuel scenario during events of snowstorms, sandstorms, droughts, and heat waves, respectively, while 514, 29, 73 and 3 hours (396, 8,18 and 0 hours) of power shortage rate higher than 2% (10%) in the zero fossil fuel scenario. In terms of controllability of Lyapunov stability, the power shortage rate can return to less than 2% (10%) within 440, 14, 8 and 0 hours (15, 3, 3, and 0 hours) in the 16% abated fossil fuel scenario during events of snowstorms, sandstorms, droughts, and heat waves, respectively, and within 503, 20, 10, and 2 hours (90, 5, 5, and 0 hours) in the zero fossil fuel scenario (Figure 4 and Supplementary Figure 9). Nevertheless, as a result of many recovery initiatives implemented by the government after weather events occurrences, we assume that variable renewable power and grid infrastructure will gradually return to normal or be repaired, indicating the power system would have an automatic recovery from weather events.

Overall, we assume Lyapunov stability in our model, i.e., for normal and extreme weather, the government has sufficient capacity to make variable renewable power and grid infrastructure gradually restore steady state to ensure electricity supply security. Also, the results show that under

normal or extreme weather, the power shortage rate can return to steady state in a limited time even if it is subject to fluctuations of more than 2%, indicating that the power system in this study can meet the Lyapunov's observability and controllability requirements.

# Supplementary Note 7 Impact of renewable energy costs on the system cost

**Impact of solar PV costs.** The sensitivity analysis of the change in solar PV cost in 2050 showed that even with a lower (higher) cost of solar PV (i.e., 48% or 28%) estimated by Chen et al [49], the total cost of a zero fossil power system would decrease by 4.8% (increase by 3.8%).

**Impact of wind power costs.** The sensitivity analysis of the change in onshore and offshore wind power cost in 2050 showed that even with a lower (higher) cost of onshore and offshore wind power (i.e., 54% and 64% or 8% and 26%) estimated by Wiser et al [48], the total cost of a zero fossil power system would decrease by 8.2% (increase by 11.9%).

**Impact of variable renewable energy and hydrogen energy storage costs.** Furthermore, we further analyzed the comparison of zero fossil fuel with long-term energy storage scenarios and 16% abated fossil fuel scenario costs and found that the total power system costs for the zero fossil fuel with long-term energy storage scenario would be comparable when variable renewable energy and long-term energy storage (hydrogen energy storage) costs are reduced by 20% and 25%, respectively (Supplementary Figure 6c)."

# Supplementary Note 8 Data sources and processing

**Wind power potential assessment.** In this study, we use real-time hourly wind speed data derived from the National Aeronautics and Space Administration (NASA) MERRA-2 dataset [80] from 1980 to 2019, with cut-in, rated and cut-out wind speeds of 3, 12, and 25 m/s, respectively, assuming a wind turbine power factor of 0.5, and an air density of 1.29 kg/m$^3$.

**Solar PV potential assessment.** The radiation intensity and air temperature for each province from 2010 to 2019 are derived from the meteorological station database using the Meteonorm software [81]. For the first method (see Equation (4)), the rated power of the solar PV panel is 300W, the radiation intensity at standard test conditions is 1000 W/m$^2$ [18], the temperature coefficient of the solar PV module is -0.35%/°C [16, 17, 18], the temperature at standard test conditions is 25 °C [18], and the shading factor of the solar PV array is 10% [16, 17, 18]. For the second method (see Equation (5)), the area of the three solar PV modules is 1.938, 1.938 and 2.83 m$^2$, respectively.

**Annual provincial electricity demand prediction.** The temperature threshold of degree-days calculation is set as 18℃. The historical electricity consumption data for each Chinese province are obtained from the China Electricity Statistical Yearbook [82], and the temperature data are obtained from the China Meteorological Data Network [83]. The historical population, GDP, and the value added of secondary industry are obtained from the China Statistical Yearbook [82]. Detailed model regression results are presented in Supplementary Table 11.

Future population growth data is derived from the United Nations Department of Economic and Social Affairs [84] and the National Population Development Plan (2016-2030) of the State Council [85]. GDP and the national ratio of the value added of secondary industry to GDP are based on previous literature [86], with corresponding provincial values calibrated using historical data. The future provincial electricity price index is assumed to be consistent with the average annual growth rate of each province from 1995 to 2019. The predicted results are summarized in Supplementary Table 12 and Table 13.

**Existing fossil fuel power plants**. The study collects plant-level data including 944 coal-fired power plants and 165 nature gas-fired power plants in 2021 obtained from the Global Power Plant Database [87], with total installed capacities of 949.1 GW and 55.8 GW, respectively, and annual $CO_2$ emissions of 3.15 Gt and 0.073 Gt, respectively.

**Supplementary Table 11. Predicted values of independent variables of the econometric model**

| Independent variable | Predicted value |
| --- | --- |
| Population | 1.38 billion people by 2050 |
| Proportion of added value of secondary industry in GDP | 25% by 2050 |
| GDP | From 5.5%-6% during the "14th five year plan" period to 2.5%-3% in 2060 |

**Supplementary Table 12. Model regression results**

| VARIABLES | FE |
|---|---|
| lnGDP | 0.930*** |
| | (42.45) |
| HDD | -0.000 |
| | (-0.80) |
| CDD | 0.000 |
| | (0.62) |
| lnSI | 0.246*** |
| | (5.23) |
| lnEPI | -0.170** |
| | (-1.99) |
| Constant | -0.138 |
| | (-0.26) |
| Observations | 748 |
| R-squared | 0.901 |
| Number of provinces | 30 |
| province FE | YES |

Note: GDP, HDD, CDD, SI, and EPI denote the GDP per capita, heating degree days, cooling degree days, the ratio of value added of secondary industry to GDP and the electricity price index, respectively. ***$p < 0.01$, **$p < 0.05$, *$p < 0.1$.

**Supplementary Table 13. National electricity demand by province in 2050**

| Province | Demand (PWh) |
|---|---|
| Beijing | 0.197487 |
| Tianjin | 0.184894 |
| Hebei | 0.853389 |
| Shanxi | 0.454643 |
| Inner Mongolia | 0.613317 |
| Liaoning | 0.527418 |
| Jilin | 0.166987 |
| Heilongjiang | 0.195607 |
| Shanghai | 0.272296 |
| Jiangsu | 1.147366 |
| Zhejiang | 1.061844 |
| Anhui | 0.508429 |
| Fujian | 0.474805 |
| Jiangxi | 0.331511 |
| Shandong | 1.247995 |
| Henan | 0.714884 |
| Hubei | 0.428012 |
| Hunan | 0.365449 |
| Guangdong | 1.375192 |
| Guangxi | 0.369227 |
| Hainan | 0.080579 |
| Chongqing | 0.229971 |
| Sichuan | 0.525654 |
| Guizhou | 0.294202 |
| Yunnan | 0.356371 |
| Shaanxi | 0.310727 |
| Gansu | 0.271335 |
| Qinghai | 0.148372 |
| Ningxia | 0.217577 |
| Xinjiang | 0.574439 |
| Tibet | 0.030084 |
| Total | 14.53 |

# Supplementary Note 9 Limitations and further perspectives

There are some limitations for our study. First, although we developed an iteration-based approach in this study by simulating a large number of cases in 2050 and obtaining the optimal power structure through cost calculation and comparisons instead of using a linear programming model with the objective of minimal cost that always aggregates the annual hours into ten or more clusters [49, 88, 89, 90]. However, it did not capture the temporal evolution characteristics of the power system configuration and did not consider more extra cases other than the simulated 10,450 cases considering the computational runtime constraints. Second, we consider the decrease in capital and operation & maintenance costs as technology advances to conform with the reality, however, like most other power system optimization models, the stranded costs for early retirement of fossil infrastructures before their end of life and the cost for curtailment of surplus wind and solar are not considered, which may underestimate the systematic costs and results in relatively low hours utilization rate of generation infrastructure and thereby a unconvincing power installation structure. Third, the slack effects of demand-side response, such as flexible charge and discharge for electric vehicle, were simply reflected by assuming a relatively low electricity-demand scenario in 2050. Fourth, to refine the model composition, we examine each province as a single node and assume that the electricity within the province can be freely transmitted. The hourly variable renewable power output is also estimated using real-time climate data in a representative city (provincial capital) and announced provincial potential of electricity generation or installed capacity. Fifth, the effects of some weather extremes, e.g., severe cold and typhoon, on the power system are not assessed due to length limit. Sixth, to ensure the emphasis of this research, long-term energy storage was not combined with the scenario of abated fossil fuel power generation with CCUS, and the impact of long-term energy storage on the power system resilience under climatic disasters was not simulated. The implications of future cost reductions of long-term energy storage, as well as the essential role that long-term hydrogen storage would play with future higher reliability standards, were briefly outlined in this study. Seventh, this study did not specify the constraints on the steady state recovery and oscillation period in the power system model, instead analyzing the Lyapunov's rule from the power shortage perspective. Finally, due to large uncertainties in future emission factors of oil usage, indirect emissions from additional oil production via the EOR process are not considered within our study boundary. These limitations will be addressed in future research.

# Supplementary References


1. Fan J-L, Xu M, Wei S, Shen S, Diao Y, Zhang X. Carbon reduction potential of China's coal-fired power plants based on a CCUS source-sink matching model. *Resources, Conservation and Recycling* **168**, 105320 (2021).
2. Fan J-L, Li Z, Ding Z, Li K, Zhang X. Investment decisions on carbon capture utilization and storage retrofit of Chinese coal-fired power plants based on real option and source-sink matching models. *Energy Economics* 106972 (2023).
3. Notice of the National Development and Reform Commission on the signing of medium- and long-term contracts for electric power in 2020. https://www.ndrc.gov.cn/xxgk/zcfb/tz/201912/t20191230_1216857_ext.html (National Development and Reform Commission, 2019).
4. In-depth report on the energy storage industry: the development of six types of energy storage and their economic assessment. https://www.vzkoo.com/document/202205111b2af51c59e3228545236eab.html (State Grid Yingda Group, 2022).
5. Li X, Zhang H, Zheng Q, Yan J, Guo y, Hu y. Electrochemical energy storage technology in energy revolution. *Bulletin of Chinese Academy of Sciences* **34**, 443-449 (2019).
6. Chen H, Ling h, Xu y. Physical Energy Storage Technology in Energy Revolution. *Bulletin of Chinese Academy of Sciences* **34**, 450-459 (2019).
7. Chen H, Liu C, Xu y, Yue f, Liu W, Yu Z. The strategic position and role of energy storage under the goal of carbon peak and carbon neutrality. *Energy Storage Science and Technology* **10**, 1477-1485 (2021).
8. Chen H*, et al.* Research progress of energy storage technology in China. *Energy Storage Science and Technology* **11**, 1052-1076 (2022).
9. Lu T, Sherman P, Chen X, Chen S, Lu X, McElroy M. India's potential for integrating solar and on- and offshore wind power into its energy system. *Nature Communications* **11**, 4750 (2020).
10. Davidson Michael R, Zhang D, Xiong W, Zhang X, Karplus Valerie J. Modelling the potential for wind energy integration on China's coal-heavy electricity grid. *Nature Energy* **1**, 16086 (2016).
11. Liu L*, et al.* Potential contributions of wind and solar power to China's carbon neutrality. *Resources, Conservation and Recycling* **180**, 106155 (2022).
12. Renewable Energy Data Sheet 2019. (China National Renewable Energy Center, 2019).
13. Kacira M, Simsek M, Babur Y, Demirkol S. Determining optimum tilt angles and orientations of photovoltaic panels in Sanliurfa, Turkey. *Renewable Energy* **29**, 1265-1275 (2004).
14. Utility-Scale Solar Photovoltaic Power Plants: A Project Developer's Guide. https://www.ifc.org/wps/wcm/connect/a1b3dbd3-983e-4ee3-a67b-cdc29ef900cb/IFC+Solar+Report_Web+_08+05.pdf?MOD=AJPERES&CVID=kZePDPG (2015).
15. Yadav AK, Chandel SS. Tilt angle optimization to maximize incident solar radiation: A review. *Renewable and Sustainable Energy Reviews* **23**, 503-513 (2013).
16. Li M, Virguez E, Shan R, Tian J, Gao S, Patiño-Echeverri D. High-resolution data shows China's wind and solar energy resources are enough to support a 2050 decarbonized electricity system. *Applied Energy* **306**, 117996 (2022).
17. Ding S, Zeng P, Xing H, Yang J, Zhou Q. A medium and long term multi-objective optimal operation method for integrated wind/PV/hydro power. *Electric Power Science and Engineering* **35**, 17-25

(2019).
18. PVWatts Version 5 Manual. (National Renewable Energy Lab. (NREL), 2014).
19. Xueli Z, Qihui L, Huimeng M, Be L. Analysis of Influencing Factors of Output Power of Photovoltaic Power Plant. *Power system and clean energy* **28**, 7 (2012).
20. Lizhen W, Hongwei T, Zhi Z, Yong L, Jin L. Evaluation of the Photovoltaic Solar Energy Potential in China Based on GIS Platform. *J Univ Shanghai for Sci Technol* **000**, 491-496 (2014).
21. China Energy & Electricity Outlook 2021. (State Grid Energy Research Institute Co., Ltd,, , 2021).
22. Ready for CCS Retrofit. https://www.iea.org/reports/ready-for-ccs-retrofit (International Energy Agency, 2016).
23. Development Roadmap of Carbon Capture, Utilization and Storage Technology in China. (Ministry Of Science and Technology, 2019).
24. Fan J-L, Shen S, Wei S-J, Xu M, Zhang X. Near-term CO2 storage potential for coal-fired power plants in China: A county-level source-sink matching assessment. *Applied Energy* **279**, (2020).
25. Kaya A, Csala D, Sgouridis S. Constant elasticity of substitution functions for energy modeling in general equilibrium integrated assessment models: a critical review and recommendations. *Climatic Change* **145**, 27-40 (2017).
26. Kuepper LE, Teichgraeber H, Baumgärtner N, Bardow A, Brandt AR. Wind data introduce error in time-series reduction for capacity expansion modelling. *Energy* **256**, 124467 (2022).
27. Heuberger CF, Rubin ES, Staffell L, Shah N, Mac Dowell N. Power capacity expansion planning considering endogenous technology cost learning (vol 204, pg 831, 2017). *Applied Energy* **220**, 974-974 (2018).
28. Zhuo Z*, et al.* Cost increase in the electricity supply to achieve carbon neutrality in China. *Nature Communications* **13**, 3172 (2022).
29. Riera JA, Lima RM, Hoteit I, Knio O. Simulated co-optimization of renewable energy and desalination systems in Neom, Saudi Arabia. *Nature Communications* **13**, 3514 (2022).
30. Glenk G, Reichelstein S. Economics of converting renewable power to hydrogen. *Nature Energy* **4**, 216-222 (2019).
31. Opportunities for Hydrogen Production with CCUS in China. https://www.iea.org/reports/opportunities-for-hydrogen-production-with-ccus-in-china (International Energy Agency, 2022).
32. Zhang S, Chen W. China's Energy Transition Pathway in a Carbon Neutral Vision. *Engineering* **14**, 64-76 (2022).
33. China's Hydrogen Energy and Fuel Cell Industry White Paper 2020. http://www.h2cn.org.cn/dynamics_detail/787.html (China Hydrogen Alliance, 2021).
34. Xiao X, Li F, Ye Z, Xi Z, Ma D, Yang S. Optimal configuration of energy storage for remotely delivering wind power by ultra-high voltage lines. *Journal of Energy Storage* **31**, 101571 (2020).
35. Yu S, Zhou S, Qin J. Layout optimization of China's power transmission lines for renewable power integration considering flexible resources and grid stability. *International Journal of Electrical Power & Energy Systems* **135**, 107507 (2022).
36. Fan J-L, Wei S, Yang L, Wang H, Zhong P, Zhang X. Comparison of the LCOE between coal-fired power plants with CCS and main low-carbon generation technologies: Evidence from China. *Energy* **176**, 143-155 (2019).
37. National electricity industry statistics for 2021. http://www.nea.gov.cn/2022-01/26/c_1310441589.htm (2021).

38. China electric coal price index. http://www.imcec. cn/zgdm. (2019).
39. About the reduction of Non-residential natural gas benchmark gate price notice. https://www.ndrc.gov.cn/xxgk/zcfb/ghxwj/201708/t20170830_960922.html?code=&state=123 (2017).
40. Ningxia-Zhejiang UHV transmission project. http://www.escn.com.cn/news/show-563970.html (Energy Storage China, 2014).
41. Projected Costs of Generating Electricity 2020. https://www.iea.org/reports/projected-costs-of-generating-electricity-2020 (2020).
42. RENEWABLE POWER GENERATION COSTS IN 2020. https://www.irena.org/publications/2021/Jun/Renewable-Power-Costs-in-2020 (2021).
43. Yang X, Nielsen CP, Song S, McElroy MB. Breaking the hard-to-abate bottleneck in China's path to carbon neutrality with clean hydrogen. *Nature Energy* **7**, 955-965 (2022).
44. Sgobbi A, Nijs W, De Miglio R, Chiodi A, Gargiulo M, Thiel C. How far away is hydrogen? Its role in the medium and long-term decarbonisation of the European energy system. *International Journal of Hydrogen Energy* **41**, 19-35 (2016).
45. Song S*, et al.* Production of hydrogen from offshore wind in China and cost-competitive supply to Japan. *Nature Communications* **12**, 6953 (2021).
46. GenCost 2020-21: Consultation draft. (CSIRO, 2020).
47. Catharina Klein. Levelized cost of energy (LCOE) per kilowatt hour of hydro energy in Japan in 2020 with forecasts until 2050. (2022).
48. Wiser R*, et al.* Expert elicitation survey predicts 37% to 49% declines in wind energy costs by 2050. *Nature Energy* **6**, 555-565 (2021).
49. Chen X*, et al.* Pathway toward carbon-neutral electrical systems in China by mid-century with negative CO2 abatement costs informed by high-resolution modeling. *Joule* **5**, 2715-2741 (2021).
50. Cost Projections for Utility-Scale Battery Storage: 2021 Update. http://www.nrel.gov/publications (National Renewable Energy Laboratory, 2021).
51. Pawluk RE, Chen Y, She Y. Photovoltaic electricity generation loss due to snow – A literature review on influence factors, estimation, and mitigation. *Renewable and Sustainable Energy Reviews* **107**, 171-182 (2019).
52. Perovich DK. Light reflection and transmission by a temperate snow cover. *Journal of Glaciology* **53**, 201-210 (2007).
53. Gruber K, Gauster T, Laaha G, Regner P, Schmidt J. Profitability and investment risk of Texan power system winterization. *Nature Energy* **7**, 409-416 (2022).
54. Shu L*, et al.* Effects of Ice Degree of Blades on Power Losses of Wind Turbines at Natural Environments. *Proceedings of the CSEE* **38**, 5599-5605 (2018).
55. Atmospheric icing of structures (ISO/TC 98/SC3). https://www.iso.org/standard/32823.html. (2001).
56. Mohammadian B, Namdari N, Abou Yassine AH, Heil J, Rizvi R, Sojoudi H. Interfacial phenomena in snow from its formation to accumulation and shedding. *Advances in Colloid and Interface Science* **294**, 102480 (2021).
57. Dong B, Jiang X, Yin F. Development and prospect of monitoring and prevention methods of icing disaster in China power grid. *IET Generation, Transmission & Distribution* **16**, 4480-4493 (2022).
58. Haeberli W, Whiteman C. Snow and ice-related hazards, risks, and disasters: Facing challenges of rapid change and long-term commitments. In: *Snow and ice-related hazards, risks, and disasters*).

Elsevier (2021).
59. IPCC, 2021: Climate Change 2021: The Physical Science Basis. Contribution of Working Group I to the Sixth Assessment Report of the Intergovernmental Panel on Climate Change. https://www.ipcc.ch/report/ar6/wg1/about/how-to-cite-this-report/ (2021).
60. Reflections on the 2008 Southern Snowstorm - Power System https://www.docin.com/p-680942535.html (2008).
61. Solaun K, Cerdá E. Climate change impacts on renewable energy generation. A review of quantitative projections. *Renewable and Sustainable Energy Reviews* **116**, 109415 (2019).
62. Li-fang A, Jin-long J. Research on Effect Characteristic of Key Parameters of PV /T Solar Photovoltaic Photothermal System. *Journal of Engineering for Thermal Energy and Power* **36**, 147-153 (2021).
63. Qi Z, Jvan Z, Zhengying X, Abudu K, Mingde Z. Design and analysis of the new energy supply system for the ecological solar greenhouse. *Northern Horticulture* 48-51 (2012).
64. Zidane IF, Swadener G, Ma X, Shehadeh MF, Salem MH, Saqr KM. Performance of a wind turbine blade in sandstorms using a CFD-BEM based neural network. *Journal of Renewable and Sustainable Energy* **12**, (2020).
65. What should we do in the face of dusty weather. http://www.cma.gov.cn/2011xzt/kpbd/Sand/2018050905/202111/t20211103_4142591.html (China Meterological Administration, 2018).
66. Khalfallah MG, Koliub AA. Effect of dust on the performance of wind turbines. *Desalination* **209**, 209-220 (2007).
67. Mongolia, northern China and other places suffered from a wide range of SDS. http://www.asdf-bj.net/publish/cms/view/acbf160566bf4363819adf51f0f63a2a.html (National Meteorological Centre Copyright, 2021).
68. 3-15 Beijing Sandstorm. https://baike.baidu.com/item/3%C2%B715%E5%8C%97%E4%BA%AC%E6%B2%99%E5%B0%98%E6%9A%B4/56314289?fr=aladdin (Baidu Encyclopedia, 2021).
69. 3-15 Ningxia Sandstorm. https://baike.baidu.com/item/3%C2%B715%E5%AE%81%E5%A4%8F%E6%B2%99%E5%B0%98%E6%9A%B4/56327848 (Baidu Encyclopedia, 2021).
70. 3-15 in Hebei Province, a wide range of sandstorm. http://hebei.hebnews.cn/2021-03/16/content_8417968.htm (2021).
71. Inner Mongolia suffered the strongest sandstorm process in 10 years. https://baike.baidu.com/reference/56358621/7458NOHPsqIwRK6u_1mhXCzSbFGwuCs2R5jMHW3SD4bmE2sYkzCmptwH4TB4zw98p8mMfIjiRCi3M7vXRKHNvbxaK0dPOZIzcqw381333jpAkONa2j-YhqcrUbU (Baidu Encyclopedia, 2021).
72. 3-15 Xinjiang Sandstorm. https://baike.baidu.com/item/3%C2%B714%E6%96%B0%E7%96%86%E6%B2%99%E5%B0%98%E6%9A%B4/56346291 (Baidu Encyclopedia, 2021).
73. Sandstorm in 11 places in Gansu. https://baijiahao.baidu.com/s?id=1694401673011840351&wfr=spider&for=pc (China Gansu Network, 2021).
74. Shanxi even issued 34 sandstorm warning news. https://new.qq.com/omn/20210316/20210316A0BVQM00.html (Tencent, 2021).

75. Liang Z*, et al.* Heat wave, electricity rationing, and trade-offs between environmental gains and economic losses: The example of Shanghai. *Applied Energy* **184**, 951-959 (2016).
76. Pérez JC, González A, Díaz JP, Expósito FJ, Felipe J. Climate change impact on future photovoltaic resource potential in an orographically complex archipelago, the Canary Islands. *Renewable Energy* **133**, 749-759 (2019).
77. Why is Sichuan, a major hydropower province, in power shortage? https://www.sc.gov.cn/10462/10464/13722/2022/8/18/a041da76a6cd45b79b9f39d89b06187d.shtml (2022).
78. Drought and flood monitoring in China. http://cmdp.ncc-cma.net/extreme/dust.php?Year=2022&Month=8&Day=12&search=%CB%A2%D0%C2%CD%BC%D0%CE (National Climate Center, 2023).
79. Electricity supply business rules. http://www.nea.gov.cn/2012-01/04/c_131262676.htm (National Energy Administration, 2012).
80. Gelaro R, Mccarty W, Suárez MJ, Todling R, Zhao BJJoC. The Modern-Era Retrospective Analysis for Research and Applications, Version 2 (MERRA-2). **30**, (2017).
81. Meteonorm. www.meteonorm.com (2021).
82. NBS. *China Electric Power Yearbook*. National Bureau of Statistics (1996-2020).
83. Hourly data from surface meteorological stations in China. http://data.cma.cn/ (National Meteorological Information Center, 2020).
84. World Population Prospects： The 2019 Revi-sion. (United Nations Department of Economic and Social Affairs, 2019).
85. Notice of the State Council on the Issuance of the National Population Development Plan (2016-2030). http://www.gov.cn/zhengce/content/2017-01/25/content_5163309.htm (China State Council, 2016).
86. Zhang X*, et al.* Research on the Pathway and Policies for China's Energy and Economy Transformation toward Carbon Neutrality. *Management World* **38**, 35-66 (2022).
87. A comprehensive, global, open source database of power plants. https://datasets.wri.org/dataset/globalpowerplantdatabase (Global Power Plant Database, 2021).
88. Zeyringer M, Price J, Fais B, Li P-H, Sharp E. Designing low-carbon power systems for Great Britain in 2050 that are robust to the spatiotemporal and inter-annual variability of weather. *Nature Energy* **3**, 395-403 (2018).
89. Daggash HA, Mac Dowell N. Structural Evolution of the UK Electricity System in a below 2°C World. *Joule* **3**, 1239-1251 (2019).
90. Bistline JET, Blanford GJ. Impact of carbon dioxide removal technologies on deep decarbonization of the electric power sector. *Nature Communications* **12**, 3732 (2021).
